# Structure-Driven Ion Transport in Conjugated Polymers with Crown Ether Side Chains: From the Nano- to Microscale

**DOI:** 10.1021/acs.chemmater.6c00382

**Published:** 2026-07-28

**Authors:** Isabelle Heinzen, Ariel Lifer, Eyal Stein, Liad Hayoun, Christina J. Kousseff, Christian B. Nielsen, Gitti L. Frey, Natalie Banerji

**Affiliations:** † Department of Chemistry, Biochemistry and Pharmaceutical Sciences, 27210 University of Bern, Freiestrasse 3, Bern 3012, Switzerland; ‡ Department of Materials Science and Engineering, Technion − Israel Institute of Technology, Haifa 32000, Israel; § Department of Chemistry, School of Physical and Chemical Sciences, 4617 Queen Mary University of London, Mile End Road, London E1 4NS, U.K.

## Abstract

In this study, five poly­(3,4-ethylenedioxythiophene) PEDOT derivatives with different 15-crown-5 contents (0–100%) are synthesized. This allows a systematic variation of nanoscale ordering, porosity, and selectivity toward sodium ions. We thus built a polymer platform to investigate the electrolyte and structural dependence of electrochemical doping. We find a trade-off in performance, where adding more crown ether enhances electronic delocalization and nanoscale ordering but suppresses ion transport because of a more compact structure with smaller pores. Spectroelectrochemistry shows that a higher crown ether content narrows the voltage range over which doping takes place (redox window width). The redox onset is shifted by the anion of the electrolyte, while the cation plays a minor role despite the chelation to the crown ether. From this systematic study, we identify PEDOT-C50 with 50% crown ether as the ideal derivative, as it combines the highest volumetric capacitance with fast switching dynamics. In chloride electrolytes, its doping remains thickness-independent, making PEDOT-C50 particularly attractive for up-scaling and bioelectronic applications.

## Graphical abstract

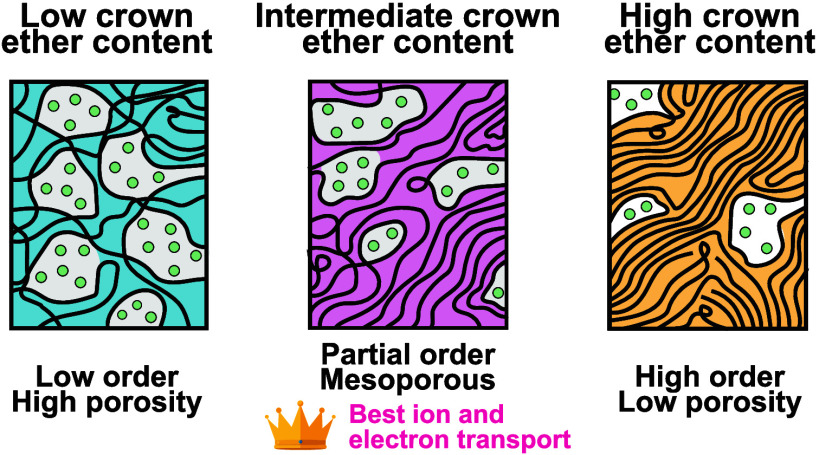

## Introduction

Organic mixed ionic-electronic conductors (OMIECs) are an emerging class of materials in the field of bioelectronics, as they enable both ionic and electronic transport throughout the bulk of the material.^1^ They combine advantageous properties such as their soft and flexible nature, versatile processing, and synthetic tunability. Therefore, they have a wide range of applications at the interface of biology and electronics, encompassing wearable to implantable devices used as sensitive biomedical sensors.^2–4^ The polymer film structure directly governs the material performance, especially the charge transport, ion permeation, and ionic transport.^5–7^ However, an in-depth understanding of the relation between the structural composition and the corresponding ion transport has not yet been developed.

Most organic semiconductor films have a semicrystalline structure with coexisting ordered and disordered domains.^8,9^ The degree of order, respectively disorder, can be fine-tuned by side-chain engineering. In various polymer systems, it has been shown that the insertion of polar ethylene glycol side chains leads to increased affinity for aqueous ions and a reduction of polymer ordering, typically favoring the ion permeation and ionic transport. However, it also limits the charge transport due to the evolution of disordered domains.^10–14^ While side chains are typically required for solution-processable OMIECs, electropolymerization offers an alternative platform for depositing systems sparse or lacking in side chains as for instance seen with the use of polypyrrole in Wrighton’s seminal work developing the organic electrochemical transistor.^15^ Electropolymerization is compatible with statistical copolymerization of similar monomer units,^16^ monomer units bearing biomarker binding sites,^17^ and its many deposition parameters can be used to tune, e.g., film thickness, morphology, and electronic properties.^18^

The choice of electrolyte also influences the OMIEC performance. For p-type polymers, anions act as counterions to stabilize the holes formed by electrochemical doping. Flagg et al. evidenced the effect of different counterions and pointed out the importance of anion chemistry within the used polymer matrix.^19^ Smaller, less polarizable anions such as chloride (Cl^–^), fluoride (F^–^), or bromide (Br^–^) are more hydrophilic and bear a large hydration shell, necessitating more polar polymers with sufficient space to transport the hydrated anions within the film. Larger anions such as hexafluorophosphate (PF_6_^–^) or trifluoromethanesulfonimide (TFSI^–^) are highly polarizable and bring little to no water into the polymer film upon doping, making them compatible with more hydrophobic polymers such as poly­(3-hexylthiophen-2,5-diyl) (P3HT).^19–23^

Although the cations in the electrolyte are often termed spectator ions during p-doping, it has been shown that the expulsion of noncompensating ions from the film can play a major role in the electrochemical doping.^24,25^ This makes it interesting to tune selectivity toward alkali cations by introducing side chains with crown ether functional groups. The size of the crown ether determines the selectivity toward the specific alkali cation. Wustoni et al. investigated the selectivity of polythiophenes bearing different crown ethers toward sodium and potassium anions under electrochemical doping conditions, as also studied by Yu et al. in a perylene diimide small-molecule system.^16,26^ Kousseff et al. additionally established a synthetic platform to control the stability and morphology of electropolymerized PEDOT-based polymer films by covalently attaching a 15-crown-5 ether moiety onto 3,4-ethylenedioxythiophene (EDOT-crown) prior to electropolymerization. The resulting films were subsequently electrochemically doped to assess their cyclic voltammetric response, stability, and doping behavior. The inclusion of the crown ether led to improved stability and a smoother surface morphology of the resulting polymer film.^27^

Here, we expand the PEDOT-crown ether system developed by Kousseff et al. to more intermediate states of crown ether functionalization by varying the feed ratio of EDOT and EDOT-crown ether during the electropolymerization. Beyond impacting the selectivity toward sodium cations, we find that tuning the 15-crown-5 content from 0 to 100% strongly affects the nanoscale polymer conformation and the microscale porosity of the films. Polymers with high porosity enable fast ion uptake and high volumetric capacitance due to their enhanced surface area.^5^ Thus, the polymer series presents a platform to investigate (i) the effect of nanoscale ordering on electrochemical doping, (ii) the role of porosity on the permeation and transport of different anions, and (iii) the impact of film affinity to the noncompensating cations on ion dynamics during electrochemical doping. Using advanced techniques such as time-resolved Vis–NIR spectroelectrochemistry, scanning electron microscopy (SEM), and electrochemical impedance spectroscopy, we unravel the doping and dedoping behavior of the PEDOT-crown derivatives in different aqueous electrolytes. We identify the PEDOT derivative with 50% crown content as optimal because of a favorable balance of chain ordering and porosity. By investigating different film thicknesses, we show that the ion transport is not limited by film thickness when an ideal affinity between the ions and the polymer matrix is established.

## Experimental Section

### Materials

Potassium hexafluorophosphate (KPF_6_, ≥99%) was purchased from Sigma-Aldrich. Potassium chloride (KCl) was purchased from Hänseler. Sodium chloride (NaCl, Ph.Eur./USP) was purchased from Schweizer Rheinsalinen. An Ag/AgCl pellet electrode (Harvard Apparatus, 1 mm diameter) was purchased from Warner Instruments. EDOT (3,4-ethylenedioxythiophene) was purchased from Fluorochem, and EDOT-crown was synthesized according to our previously reported method.^27^

### Electropolymerization

For the electropolymerization on indium–tin oxide (ITO) covered glass substrates, the substrates were cleaned with detergent, sonicated in water (3 × 15 min), acetone (2 × 10 min) and isopropanol (2 × 5 min), and plasma cleaned for 5 min right before use. The electropolymerization was performed by chronoamperometry at 1.2 V for up to 45 s (depending on desired film thickness, see Table S1) at ambient conditions from 10 mM monomer solution in degassed acetonitrile with 0.1 M TBAClO_4_ as the supporting electrolyte. The crown ether content in the polymer film was controlled by varying the molar ratio between the two monomers, EDOT and EDOT-crown, with the total monomer concentration being 10 mM. For example, PEDOT-C25 was prepared using a 3:1 molar ratio of EDOT and EDOT-crown.

### Sample Preparation

For the Vis–NIR spectroelectrochemistry measurements, precision tip cotton swabs were used to partially remove the polymer film to define an area of 12 mm^2^ (3 mm × 4 mm) on the ITO substrate. The film thicknesses were measured using white-light interferometry (Contour GT, Bruker): a complete overview of the sample thicknesses is given in Table S1. The samples were placed in a home-built spectroelectrochemical cell with both the Ag/AgCl electrode and various electrolyte solutions.

For scanning electron microscopy, films were prepared by electropolymerization on highly doped Si++ substrates. These substrates were first coated via thermal evaporation with a 10 nm Cr adhesion layer, followed by a 100 nm Au layer.

For impedance measurements, ultraflat quartz-coated glass substrates were cleaned in an ultrasonic bath in acetone, methanol, and isopropanol (15 min each) and blow-dried in N_2_ (99.995%). Then, substrates were placed in an OFET shadow mask (E291, Ossila Ltd.) but without the fine feature of the transistors, thereby only creating a pattern of 2 identical 1 mm^2^ squares with a lead wire between them. The assembly was put inside a metal thermal evaporator (Edwards Auto 500), where 5 nm Cr and 50 nm Au were evaporated at a rate of 0.03–0.06 nm s^–1^ at a pressure of ∼10^–6^ Torr. After the evaporation of metal contacts, the substrates were sonicated for 15 min in isopropanol and blow-dried in N_2_. Then, the leading wires were masked with Kapton tape and 1 mm^2^ pads were electropolymerized with PEDOT in the EDOT bath.

### Vis–NIR Spectroelectrochemistry

The Vis–NIR spectroelectrochemistry was performed using a home-built setup.^28^ The setup consists of a halogen light source (HL-2000, Ocean Insight), a Flame UV–Vis and a Flame NIR spectrometer (Ocean Insights), which are triggered by a digital delay/pulse generator (DG535, Standford Research Systems). A LabVIEW code was used to operate the instruments and a data acquisition card (USB-6008, National Instruments) to measure the voltage between the electrodes. For the time-resolved Vis–NIR spectroelectrochemistry, square voltage pulses were applied from −0.8 to 0.8 V (Δ*V* = 0.1 V), with a dwell time at each step between 5 and 15 s to ensure complete electrochemical conversion (after this time, the steady-state absorbance was taken). Both the transient spectra and the transient response current (converted to voltage using a low-noise current preamplifier (SR570, Stanford Research Systems)) were measured with the same data acquisition card (USB-6008), with a time resolution of 10 ms. The intrinsically doped films were dedoped at positive voltages and further doped at negative voltages. Between each voltage step, a subsequent voltage doping step was included by applying −0.4 V for 5 to 15 s. The electrolyte solutions were 0.1 M NaCl, 0.1 M KCl, and 0.1 M KPF_6_ in distilled water. The electrolyte solution was degassed with nitrogen gas for 15 min prior to the measurements.

### Scanning Electron Microscopy

Film morphology was characterized using a Zeiss Ultra-Plus FEG-SEM instrument at an accelerating voltage of 1.5 kV. Top-view images to resolve the surface topography were acquired using an in-lens secondary electron (SE) detector. For extracting the area-averaged size of the pores, the high-resolution SEM micrographs were analyzed using intensity thresholding to classify pixels as either pore or PEDOT-crown matrix. The resulting binary images were processed in MATLAB using *regionprops* to quantify pore areas and their self-weighted means. These values were then converted to physical units by using the image scale bar.

### Impedance Spectroscopy

The impedance spectra were recorded with a potentiostat (PalmSens4, PalmSens BV) in a 3-electrode setup using a 0.1 M NaCl solution, Ag/AgCl pellet as reference electrode (RE), and Pt wire as counter electrode (CE), at a frequency range between 100 kHz and 0.1 Hz. The measurements were performed at DC voltage E_DC_ = 0 V and AC amplitude of 10 mV. After measurement, the serial capacitance was calculated as $C_{s}=\frac{1}{2\pi {\mathrm{fZ}}^{\prime \prime }}$ , where *Z*″ is the imaginary part of the capacitance and f is the frequency, and the value of *Z*″ was extracted at 0.1 Hz. Then, the serial capacitance *C*_s_ was normalized by the film volume to determine volumetric capacitance *C*_s_*.

### Grazing Incidence Wide-Angle X-ray Scattering (GIWAXS)

GIWAXS was performed in a Xenocs Xeuss 3.0 apparatus equipped with a Cu Ka source (*l* = 1.54 Å^–1^) in parallel collimation, and an Eiger 2R 1 M detector. Sample to detector distance was kept at 125 mm and the incidence angle at 0.2°. 2D diffractograms were collected using 300 s of exposure per frame. All measurements were carried out in vacuum to minimize air scattering. Wedge correction and linecut integrations were performed using XSACT software by Xenocs.

## Results and Discussion

### Effect of Crown Ether Content on Nanoscale Ordering and Film Porosity

In this study, we investigate five different PEDOT derivatives that vary in the amount of 15-crown-5 content from 0 to 100%, in steps of 25% (Figure 1a). A complete sample overview, including the used film thicknesses, is shown in Table S1. The Vis–NIR absorbance of the polymers is measured for the films that were electrochemically dedoped in electrolyte (0.1 M NaCl) at an applied voltage of 0.8 V, showing the absorbance band of neutral polymer chains (Figure 1b). A clear trend in the nanoscale polymer chain ordering is apparent as the neutral absorbance band gets more structured with increasing crown ether content. PEDOT-C0, without crown ether incorporation, displays a broad structureless peak at 617 nm, indicative of torsionally disordered polymer chains with a broad distribution of conjugation lengths and poor packing into an amorphous polymer phase.^8^ As the crown ether moiety is introduced, a narrowing of the neutral band can be observed, as well as a shift toward higher wavelength to 628 nm for PEDOT-C50 (50% crown ether content) and to 639 nm for PEDOT-C100 (100% crown ether content). Concurrently, vibronic features appear with PEDOT-C100 showing peaks at 600 nm (0–1 transition) and 639 nm (more-pronounced 0–0 transition). This red shift, narrowing, and vibronic structure is typical for more ordered planar polymer chains with a more homogeneous distribution of chain segments with longer conjugation length.^29^

**Figure 1:**
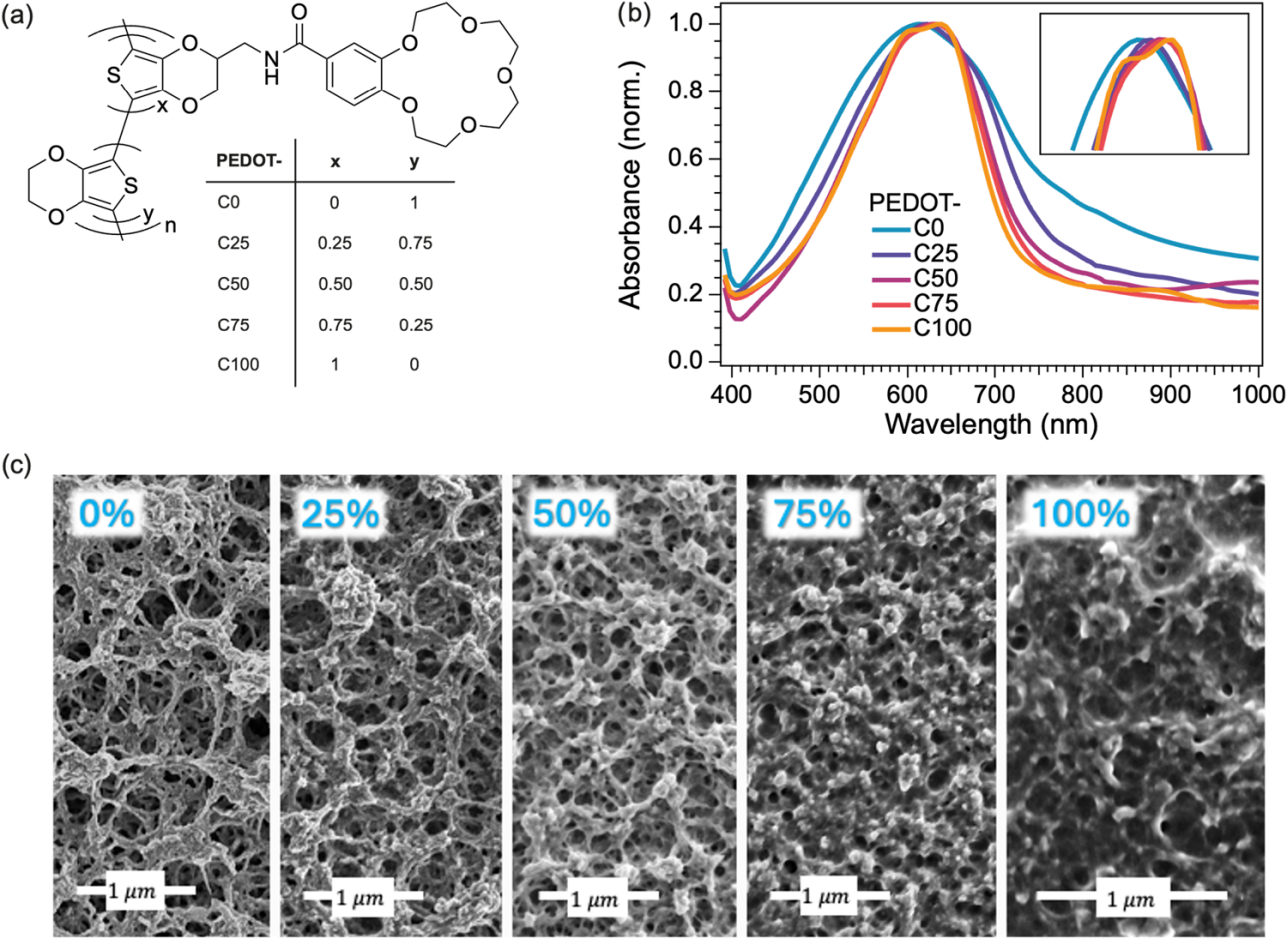
(a) Chemical structure of the PEDOT-crown polymers comprising crown-functionalized EDOT moieties (*x*) and nonfunctionalized EDOT moieties (*y*). (b) Absorbance spectra of the neutral species for the ∼100 nm dedoped PEDOT-crown derivatives. (c) SEM images showing the different microstructures of the polymer series from PEDOT-C0 (0%, left) to PEDOT-C100 (100%, right) from the top view with thicknesses ∼450 nm.

We carried out grazing incidence wide-angle X-ray scattering **(**GIWAXS). The 2D diffractograms and linecuts (Figure S1) reveal an isotropic film for PEDOT-C0 with the lamellar reflection (100) centered at q = 0.46 Å^–1^ and π–π stacking (010) at 1.83 Å^–1^. However, all films introducing the PEDOT-crown moiety display glassy GIWAXS patterns, with no diffraction peaks. These results seem to contradict the trend observed in the absorption spectra. Nevertheless, spectroscopic data reveals information related to conformational order, i.e., backbone planarity, while GIWAXS reveals periodic molecular packing. Hence, while the introduction of the bulkier PEDOT-crown disrupts the crystallinity, it enhances backbone planarity and nanoscale conformational order.^30^

At the longer length scale, we performed scanning electron microscopy (SEM) on the PEDOT-crown films to characterize their microstructures and porosities. In Figure 1c, the top-view SEM images show a reduction in pore size at higher crown ether content as well as a smoothening of the surface, which is also supported by the side-view images in Figure S2. The cross-sectional views reveal that the fibrous or dense character of the films persists throughout the full thickness of the polymer layer, indicating that these structural differences are not confined to the surface but instead reflect changes in the bulk morphology governed by the comonomer ratio. PEDOT-C0 exhibits a highly porous, fibrous network with a rough surface, whereas PEDOT-C100 displays a dense, continuous morphology with minimal observable voids. These findings indicate that increasing crown ether content not only enhances nanoscale ordering but also drives a transition toward a denser, less porous film structure.

### Electrochemical Doping of the Ordered and Disordered Polymer Chains

To investigate the impact of the nanostructure on the doping and dedoping processes, spectroelectrochemistry was carried out in the NaCl electrolyte. The PEDOT-crown polymers were deposited on conductive indium tin oxide (ITO) substrates with a thickness of roughly 100, 200, and 400 nm. For now, we discuss the films of about ∼100 nm thickness, while thicker films are described in the last section of the manuscript. SEM images for different electropolymerization durations to produce the different film thicknesses are depicted in Figure S3. The films are doped in aqueous 0.1 M NaCl electrolyte, while applying square voltage pulses ranging from −0.8 to 0.8 V, with steps of 0.1 V. A dwell time of 5–15 s was applied to ensure that the spectra in each polymer/electrolyte system had stabilized.

In Figure 2a, the steady-state absorbance spectra at different applied voltages are shown for PEDOT-C100. At the most positive voltages, the polymer is significantly dedoped and mainly the neutral absorbance (with the characteristic vibronic structure of PEDOT-C100) is seen mixed with a broad contribution of singly charged species (about 30%). Below 0.3 V, the neutral species at 639 nm is depleted, with a simultaneous growth of additional singly charged species at 989 nm (often called polarons). At more negative voltages, the increase of a broad absorbance at 1450 nm is observed, which is attributed to doubly charged species (often called bipolarons or polaron pairs). Below 0.0 V, there is a decrease of the singly charged absorbance feature as they are converted into doubly charged species.^27^

**Figure 2:**
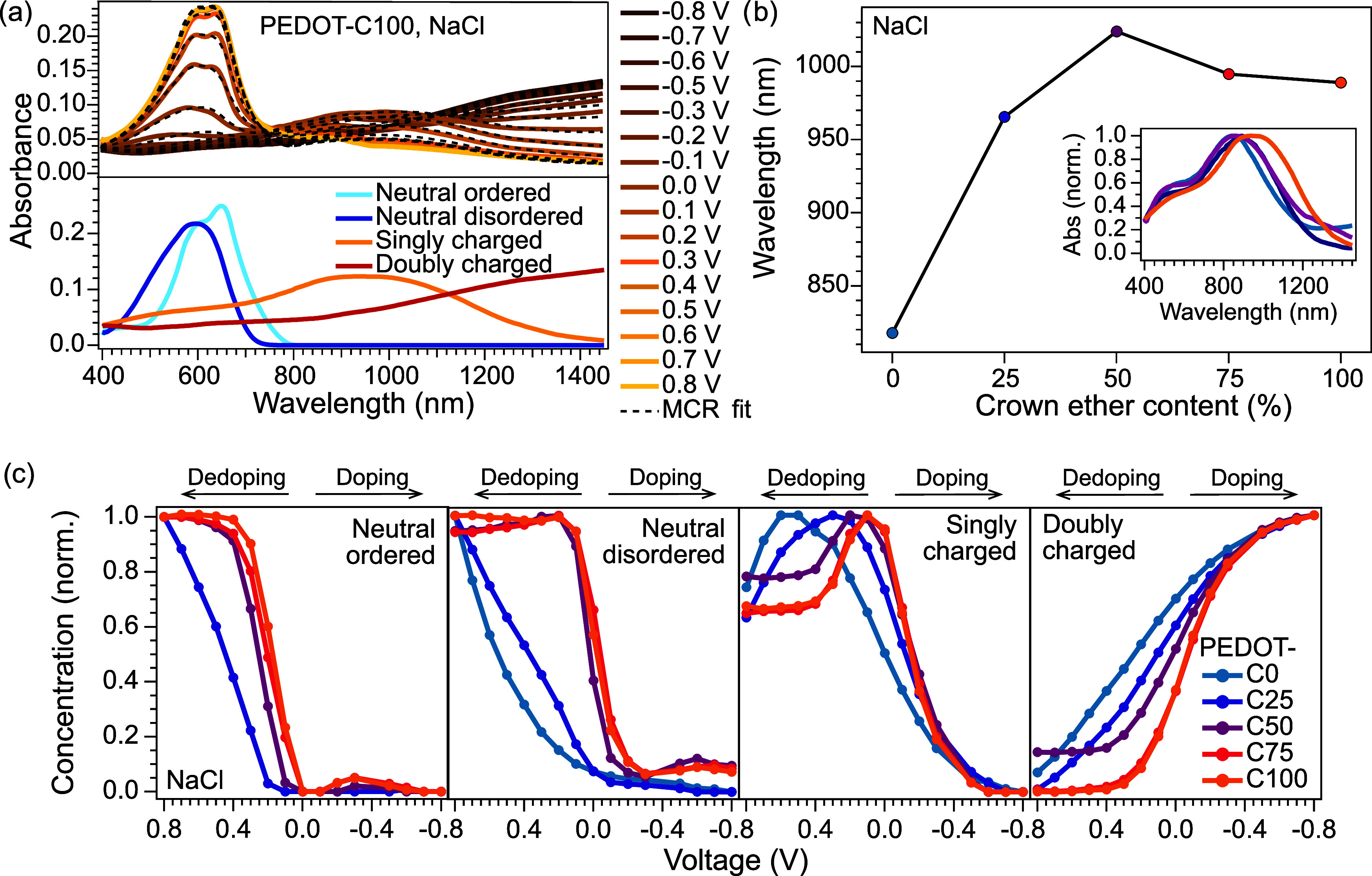
(a) Top: Steady-state absorbance spectra of the ∼100 nm thick PEDOT-C100 film in 0.1 M NaCl (aq.) upon the application of voltages ranging from 0.8 to −0.8 V (Δ*V* = 0.1 V). The dashed lines represent the fits from the MCR analysis. Bottom: Spectral signatures obtained from the MCR analysis of the different PEDOT-C100 species. (b) Shift of the maximum of the singly charged species band for the doped films with different crown ether content (as measured, without MCR). The inset shows the spectral signature from MCR for the singly charged species, additionally highlighting the shift in wavelength. (c) Normalized concentration as a function of voltage for the neutral ordered, neutral disordered, singly charged, and doubly charged species obtained by MCR for the PEDOT-crown polymers in 0.1 M NaCl (aq.).

The spectroelectrochemistry of the other PEDOT-crown derivatives follows a similar trend (Figure S4). However, the doping process is less abrupt for the polymers with less crown ether content, and the transition from singly charged to doubly charged species is more washed out. In PEDOT-C0, singly and doubly charged species seem to emerge almost concurrently. We previously showed for P3HT that ordered polymer chains preferentially form singly charged species, whereas disordered regions favor doubly charged species.^31^ This aligns with our observation that PEDOT-C0 is less ordered and therefore rather forms doubly charged species, with a less pronounced formation of distinct singly charged species.

Multivariate curve resolution (MCR) analysis was applied to the Vis–NIR spectra to deconvolute the spectral signatures of the different species (neutral ordered, neutral disordered, singly charged, and doubly charged) and to determine their concentration at the applied voltages (details in Section S4). The spectral signatures for PEDOT-C100 are shown in Figure 2a. For the other polymers, the species are slightly adapted to fit the spectroelectrochemical absorbance spectra (Figure S5). In particular, the absorbance band of the neutral disordered polymer chains is broader at a low crown ether content. For PEDOT-C0, the neutral-ordered species is absent. This again underlines the increased ordering observed with a higher crown ether content. Furthermore, both charged species display a red shift with increasing content of crown ether. This indicates that the PEDOT polymers with crown ethers have a longer effective conjugation length with more delocalized singly and doubly charged species compared to PEDOT-C0.^27^ The singly charged species for PEDOT-C0 has an absorption maximum at 818 nm, which substantially shifts to 1024 nm in PEDOT-C50 (Figure 2b). Above 50% crown ether, there is not a drastic change anymore; however, the spectrum slightly blue-shifts again to 989 nm for PEDOT-C100.

We compare the MCR concentrations of the four species as a function of applied voltage for the different PEDOT-crown polymers in 0.1 M NaCl (Figure 2c). As the MCR analysis does not provide absolute concentrations, the traces were normalized for each species by setting the maximum concentration to 1. This allows better comparison between the species evolution with voltage but does not directly compare the absolute doping level among the polymers. The spread of the voltages over which the doping and dedoping respectively occurs, which we refer to as “redox window width,” is strongly affected by the crown ether content, as this impacts the distribution of conjugation lengths in the polymer films. It is known that shorter and more-disordered polymer segments require a higher (in our case more negative) oxidation potential in order to be doped. This is consistent with the distinct doping onset of ordered and disordered domains observed in previous reports on morphology-dependent redox behavior in conjugated polymer films.^31–34^ For the neutral ordered species, there is a small dispersion in the conjugation length and therefore a narrow spread of doping energies. We find a sharp redox window, with overlapping species evolution for PEDOT-C50, -C75, and -C100. Only for PEDOT-C25 (which hardly contains any ordered chains), the redox window is broader, and no ordered neutral species are detected in PEDOT-C0. Concerning the disordered neutral species, their evolution coincides again for PEDOT-C50, -C75, and -C100 but is shifted to more negative potentials compared to the ordered neutral species. There is a clear broadening of the redox window when decreasing the crown ether content in PEDOT-C25 and -C0, as the energy required for the doping process is more heterogeneously spread in the highly disordered PEDOT chains.

All PEDOT derivatives start with a considerable concentration of singly charged species, which corresponds to 25–35% of the total species density at 0.8 V, showing that the polymers cannot be completely dedoped. The further rise of the singly charged species from neutral oxidation also reflects the broadening of the redox window in the more disordered films (Figure 2c). A similar trend occurs for the conversion of singly to doubly charged species (seen in the decay of the former and rise of the latter during doping), whereby the PEDOT-C75 and -C100 curves overlap and show the sharpest redox switching. We note that the remaining offset of doubly charged species between 0.8 and 0.4 V in PEDOT-C50 is likely an artifact of the MCR analysis. Interestingly, even though the redox window width varies between the polymers, this reflects mainly in the onset of the oxidative doping (going toward negative voltages), while the onset of the reductive dedoping (from doubly to singly charged and then to neutral species when going toward positive voltages) is always at the same voltage for all of the PEDOT-crown polymers.

### Interplay of Nano- and Microstructure in the Electrochemical Performance

We now correlate the findings about the nanostructure realized from the absorbance spectra with the microstructure observed by SEM and the volumetric capacitance values calculated from electrochemical impedance spectroscopy measurements (Figure 3). We assess the order in the films using the absolute absorbance area of the neutral ordered and disordered species, by integrating the deconvoluted MCR bands at a voltage where the films are most dedoped at 0.8 V in 0.1 M NaCl. Analysis of the neutral peak area in the most dedoped state (at 0.8 V) confirms the trends in the nanoscale film order: for PEDOT-C0, there are only disordered neutral species; for PEDOT-C25, the neutral disordered is still dominant but some neutral ordered states are apparent; and for PEDOT-C50, the neutral ordered and disordered are present in almost equal shares, while both PEDOT-C75 and -C100 possess more ordered than disordered neutral species with a ratio around 1.5 (∼60% ordered species) (Figure 3a).

**Figure 3:**
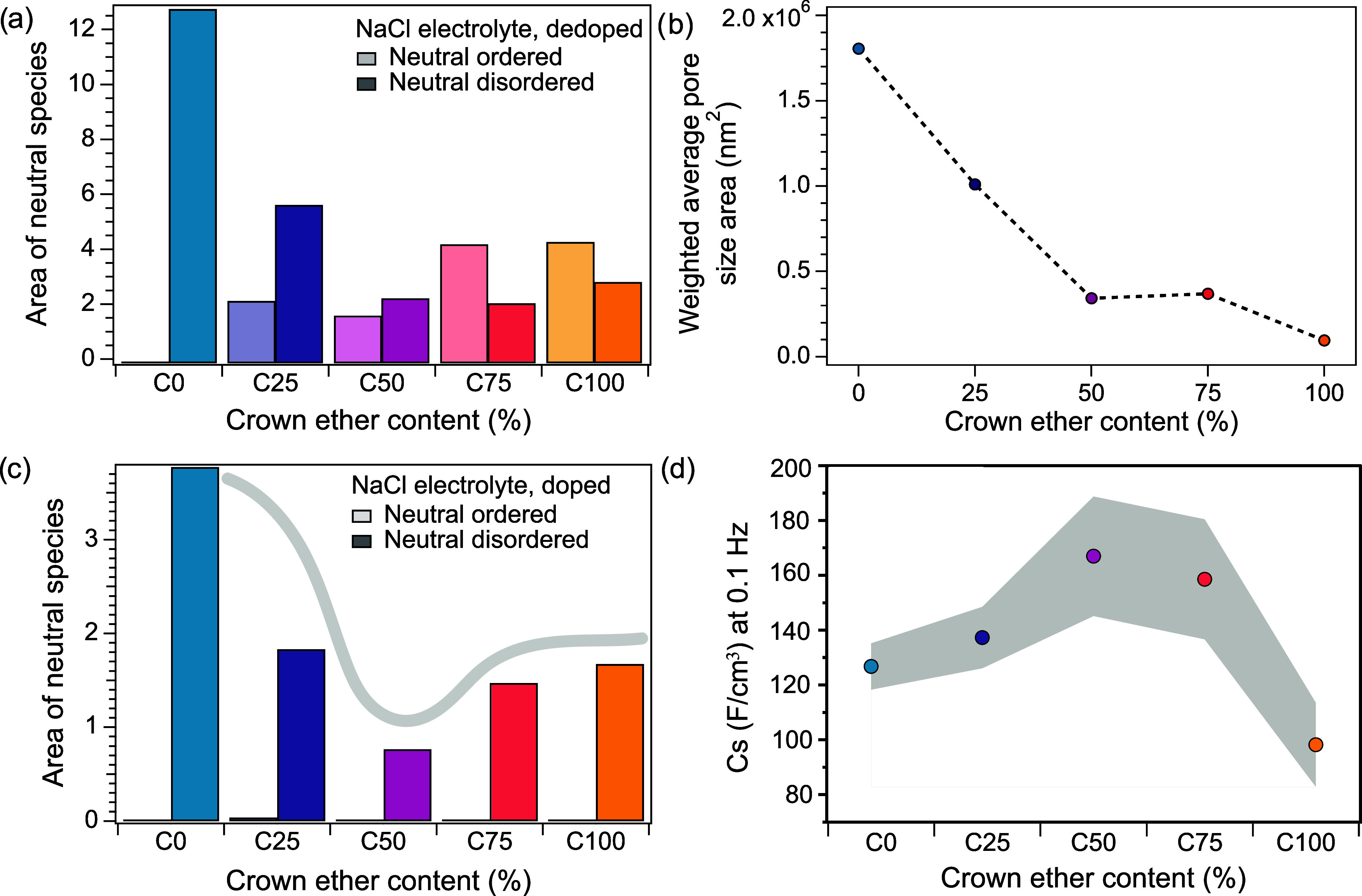
(a) Area of the deconvoluted spectral bands of the ∼100 nm films corresponding to ordered and disordered neutral species from MCR analysis in the most dedoped films (at 0.8 V). The darker color corresponds to the disordered neutral and the lighter color to the ordered neutral species. (b) Area-averaged size of the pores for the different PEDOT-crown derivatives of ∼450 nm films extracted from the SEM images. (c) Area of the deconvoluted neutral ordered and disordered species in the doped films of ∼100 nm (at 0.4 V for PEDOT-C0, 0.2 V for PEDOT-C25 and 0.0 V for PEDOT-C50, -C75, and -C100; this is the voltage where the maximum absorbance of neutral/singly charged species = 1). The gray line shows the trend in the amount of remaining disordered neutral species. (d) Volumetric capacitance for the different PEDOT-crown derivatives (∼450–700 nm thickness). All of the measurements in this figure were carried out in 0.1 M NaCl (aq.).

We then evaluated the microstructure by calculating the weighted average pore size from the SEM images in Figure S6. This calculation (see Section S5 for details) shows a decrease of the area-averaged pore size with increasing crown ether content. Importantly, the total pore area follows an almost perfectly linear decrease (*R* ^2^ = 0.99), emphasizing the quantitative predictability of the morphological evolution and the modular tunability of the film structure. Similar to the red shift of the charged species absorbance due to increased delocalization, we also see a large difference going from PEDOT-C0 to C50 in terms of porosity, while a higher crown ether content (C75 to C100) induces only minor changes compared to those of PEDOT-C50 (Figure 3b). Correlating the trends for the nano- and microstructure, we can conclude that the films become more conformationally ordered and simultaneously display a smoother and less porous microstructure with increasing crown ether content. This leads to a counterintuitive morphological transition, where PEDOT evolves from an open, fibrous network to a dense and compact morphology with the addition of the bulky crown ether. According to the GIWAXS data, the insertion of the bulky side groups still causes disruption of crystalline intermolecular packing, but the crown ether side chains promote backbone planarization,^27^ resulting in locally more ordered and compact films. The densification is supported by the optical spectra in Figures 1b and 2b, where the progressive narrowing and the red shift of the absorbance indicate increased effective conjugation length and backbone planarity. These electronic structure changes enable tighter microstructural packing despite the bulky side chains, with PEDOT-C50 reaching an optimal degree of order before excessive densification begins to hinder ion transport, as observed for PEDOT-C100.

To quantify the effect of nanoscale order on the doping process, the area of the ordered and disordered neutral species is compared in the polymer films at a comparable doping level where the absorbance ratio between the neutral species and the singly charged species is around one (between 0.4 and 0.0 V, Table S2). For all of the PEDOT-crown polymers, the area of the ordered neutral species is almost completely depleted at this level, as the ordered polymer chains are oxidized at more positive voltages than the disordered ones (Figure 3c). In PEDOT-C0, the highest amount of neutral disordered species remains upon doping; however, it is still 3.4 times lower than that in the dedoped film. PEDOT-C25, -C75, and -C100 have a comparable amount of neutral disordered polymer left after doping, even though for PEDOT-C25 less than half of the original neutral disordered species are left, whereas, for PEDOT-C75 and -C100, the amount of neutral disordered is similar in the dedoped and doped states. This is due to them initially containing more neutral ordered species that are preferentially doped, while the neutral disordered population stays almost untouched. The neutral disordered species is most depleted for PEDOT-C50, where only one-third of its original amount remains after doping even though the neutral ordered and disordered species have an equal share in the dedoped state.

The gray line in Figure 3c showcases the trend observed in the amount of remaining neutral disordered species upon doping, which is reciprocal to the trend in the volumetric capacitance, where a maximum for PEDOT-C50 is observed (Figure 3d). The volumetric capacitance was obtained by electrochemical impedance spectroscopy in 0.1 M NaCl (Figure S7). A high volumetric capacitance requires both efficient ion transport and available electronic states for charge storage. Here, PEDOT-C50 can store most charges per volume, and even the disordered neutral species is depleted to the highest extent. We demonstrate a central trade-off in OMIEC performance, where adding more crown ether enhances electronic delocalization and nanoscale ordering but suppresses ion transport because of a more compact structure with smaller pores. In contrast, less crown ether facilitates ion movement due to larger pores, but ordering is lost, leading to less complete doping of the disordered polymer chains, which require higher oxidation potential to be doped and likely less good electronic transport. PEDOT-C50 shows the best balance between these competing effects, which is reflected in its maximum volumetric capacitance. At the extremes, PEDOT-C0 is limited by disorder, while PEDOT-C100 is limited from restricted ion transport caused by the densified film structure.

### Electrolyte: Variation of the Cation and Anion Nature

To generalize the structure-dependent trade-off, we tested the same PEDOT series using three different aqueous electrolytes with varying anions and cations. We chose 0.1 M NaCl, 0.1 M KCl, and 0.1 M KPF_6_, which are common in OMIEC material studies and share either the same anion or cation, in order to distinguish their effect. We focus again on films of ∼100 nm thickness, and the spectroelectrochemistry is shown in Figures S4, S8, and S9. The nanostructure in those films is not significantly affected by the electrolyte, as shown by a comparable ratio of neutral ordered to disordered species (Figures S16 and S17) and by a similar trend in the peak position of the singly charged species (Figure S19).

We compare the evolution of the normalized species concentration as a function of voltage (from MCR analysis) and see a similar trend among the five polymers in the three electrolytes (Figure S20). As illustrated in Figure 4a for PEDOT-C100, both KCl and NaCl electrolytes display almost the same redox onset and redox window width in both the doping and dedoping directions for all four involved species. Here, the Cl^–^ anion, which is primarily involved in the doping process as it compensates the positive charges formed on the polymer backbone, is the same for both electrolytes. However, the cation (K^+^ or Na^+^) varies, and it has been shown that the expulsion of noncompensating ions from the film can play a major role in the electrochemical doping.^24,25^ The 15-crown-5 ether is selective toward Na^+^ due to the cavity size matching with the ionic radius, although sandwich-type interactions can lead to other cation-specific interactions with K^+^.^16,26,35,36^ If noncompensating ions affect the doping process, we would expect a different behavior in KCl and NaCl electrolytes when the affinity of the film toward the cations is tuned by increasing the crown ether content.^24,25^ However, no change due to the nature of the cation and the accompanying chelating property of the crown ether is detected, showing that the noncompensation ion plays a minor role here.

**Figure 4:**
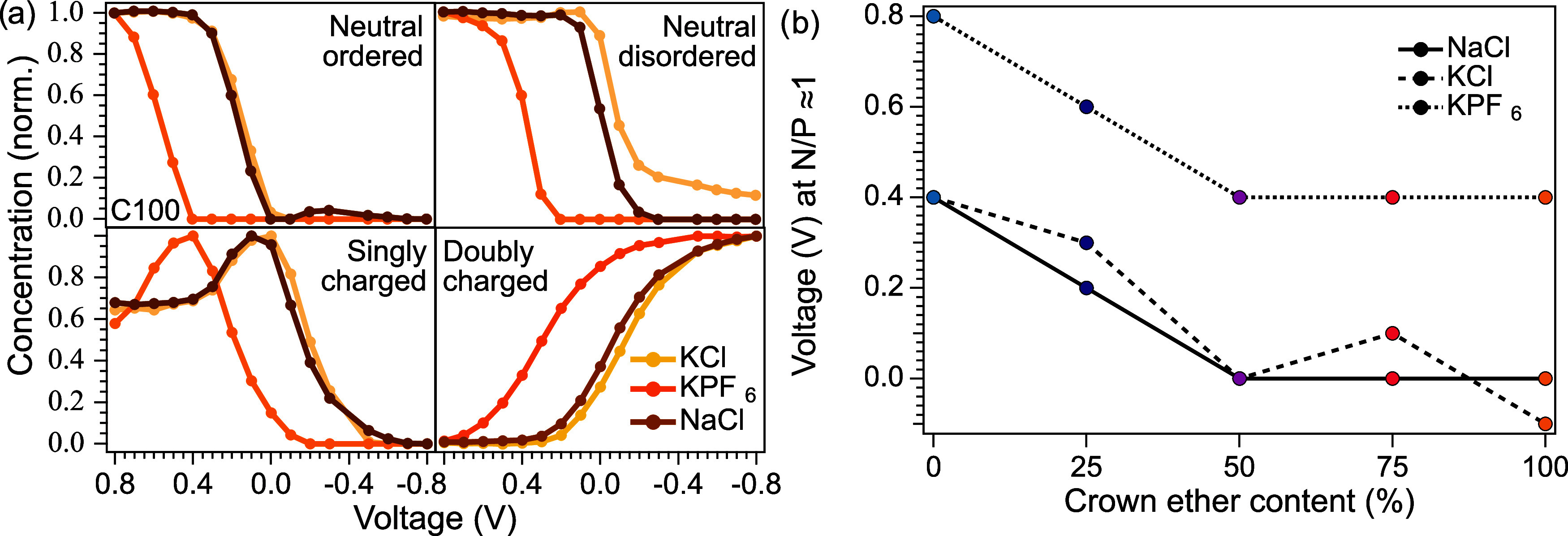
(a) Normalized MCR species concentration as a function of voltage (from 0.8 to −0.8 V) for the neutral ordered, neutral disordered, singly charged, and doubly charged species for PEDOT-C100 in the different electrolytes. (b) Voltages applied to reach a comparable doping level (where the ratio of neutral (N) to singly charge species (SC) absorbance equals 1) for all PEDOT-crown derivatives in 0.1 M NaCl, KCl, and KPF_6_ (aq.).

In contrast, in the KPF_6_ electrolyte, the curves for PEDOT-C100 are shifted by about 0.4 V to more positive voltages compared to KCl and NaCl (Figure 4a). This shows that the onset of the oxidation, respectively reduction, of PEDOT-crown polymers is determined by the compensating anion (Cl^–^ or PF_6_^–^). A similar behavior was reported by Flagg et al. for P3HT, which underlines that this is a common feature of hydrophobic polymers. As Cl^–^ is surrounded by a large hydration shell, the ion permeability into the film is less favorable and requires a higher (more negative) oxidation voltage, while the smaller hydration shell of PF_6_^–^ favors the doping process. Thus, the different behavior with the PF_6_^–^ counterion can be related to different interactions taking place between the anion and the polymer matrix. For PEDOT-C0 and -C25, the shift between the redox curves of the neutral species is less pronounced due to better transport of hydrated Cl^–^ with the larger pores, which compensates for the higher affinity of the less hydrated PF_6_^–^ anion to the more hydrophobic side chains (Figure S20). On the other hand, the denser film structure of PEDOT-C100 favors the transport of PF_6_^–^ (no large hydration shell), causing an important shift. In spite of the different oxidation onsets, the use of different anions does not have any influence on the redox window width as this is purely determined by the ordering of the polymer itself.

On the one hand, we have a change of redox onset when working in different electrolytes and on the other hand, a change of redox window width when varying the PEDOT-crown derivatives and their structural ordering. Therefore, the PEDOT-crown films doped to a different extent under the application of the same voltage. To achieve a comparable doping level, we apply again a voltage where the ratio of neutral to singly charged species absorbance is around one (Figure 4b and Table S2). This doping level is reached earlier (at more positive voltages) for PEDOT-C0 in all electrolytes, which is likely related to its larger pore structure. Then, gradually higher oxidative (less positive) voltages need to be applied, and from PEDOT-C50 on, no discernible differences are observed. Again, the KCl and NaCl electrolytes show similar behavior, while KPF_6_ is shifted by 0.4 V for all of the polymers.

### Dedoping Dynamics

To determine the dedoping dynamics of the different polymer films, we measured the time-resolved absorbance spectra with a resolution of 10 ms while switching the voltage from −0.4 V (strongly doped state, mainly doubly charged species) to either a comparable dedoping level (at variable voltage) or to the same voltage of 0.8 V (at variable dedoping level). Together with MCR analysis, this allows us to investigate the dynamics of each species separately. Figure 5a shows the normalized dynamics of the neutral disordered rise during dedoping of the five PEDOT-crown derivatives in the 0.1 KPF_6_ electrolyte to a comparable level, where the ratio of neutral to singly charge species absorbance equals 1 (voltage indicated in the legend). PEDOT-C0 and -C25 show similar fast dynamics, whereas, from PEDOT-C50 onward, the process gradually slows down, with PEDOT-C100 being the slowest. This suggests that increasing the crown ether content in the PEDOT polymers systematically reduces the dedoping rate. This effect is attributed to the different voltage steps required to reach comparable dedoping levels. For PEDOT-C0 and -C25, a larger voltage step (Δ*V*_C0_ = 1.2 V, Δ*V*_C25_ = 1.0 V) is needed, while PEDOT-C50, -C75, and -C100 reach a similar dedoped state with a smaller voltage step (Δ*V*_C50.75.100_ = 0.9 V). Since the applied potential difference provides the driving force for the redox reaction and ion transport, a smaller voltage step directly translates to slower dedoping dynamics.^37,38^

**Figure 5:**
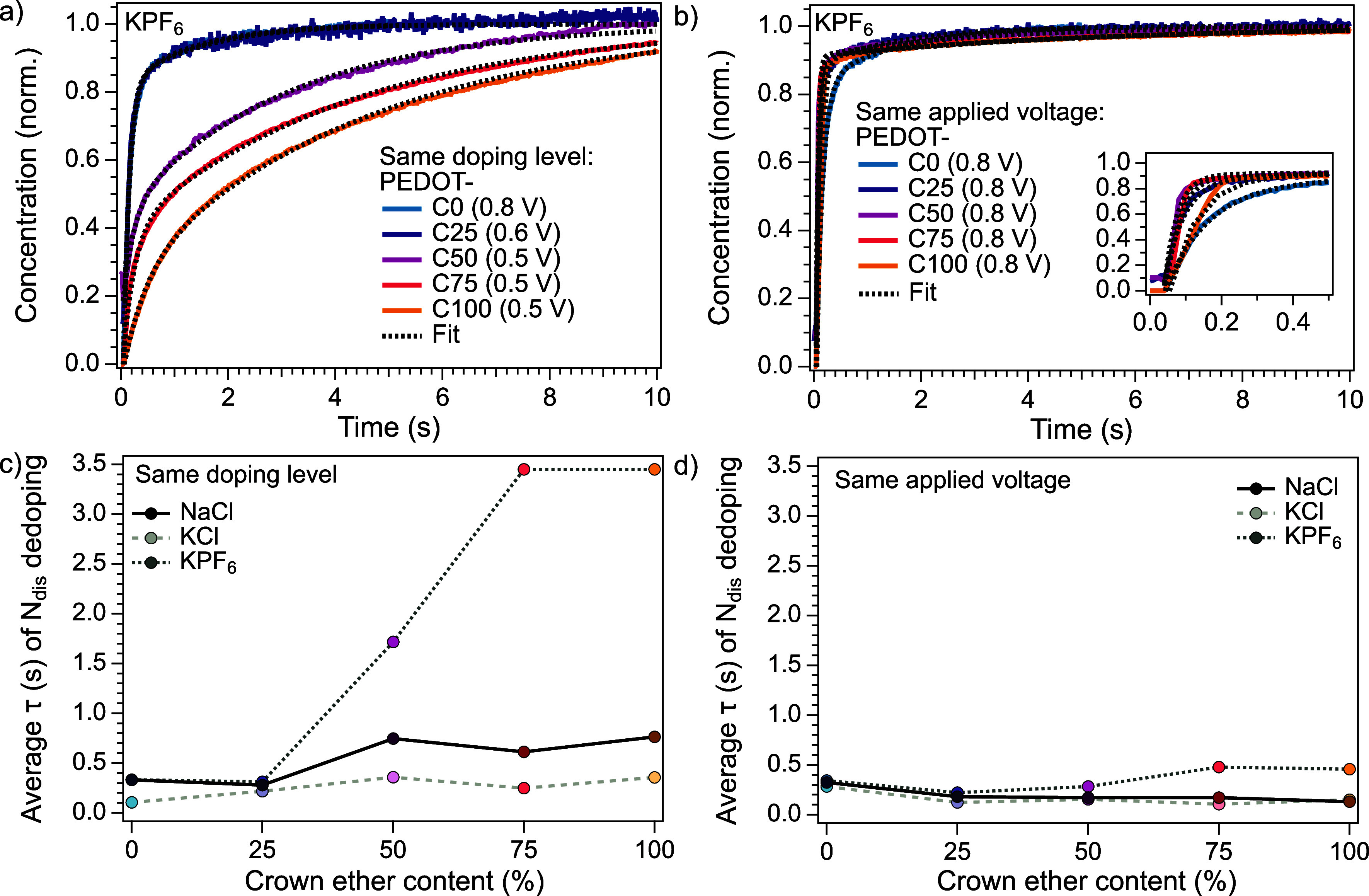
(a, b) Comparison of time-resolved normalized concentration of neutral disordered species for the different PEDOT-crown derivatives (∼100 nm films) in 0.1 M KPF_6_ (aq.) for (a) a dedoping step from −0.4 V to a similar dedoping level (voltage indicated in the legend) and (b) the same applied voltage step from −0.4 to 0.8 V. (c, d) Average time constants from a biexponential fit for the neutral disordered dedoping, comparing the PEDOT-crown derivatives in the three different electrolytes 0.1 M NaCl, KCl, and KPF_6_ (aq.) for (c) a dedoping step from −0.4 V to a similar dedoping level, (d) a voltage step from −0.4 to 0.8 V.

Therefore, the lower driving force in the more crown-functionalized derivates explains their progressively slower dedoping dynamics. To eliminate the effect of the driving force, the normalized dynamics are also compared using the same voltage step (Δ*V* = 1.2 V) to dedope the polymer films in the KPF_6_ electrolyte (Figure 5b). In this case, the PEDOT-crown derivatives are all doped by applying −0.4 V and subsequently dedoped (to a variable extent) at 0.8 V. No significant difference is observable in this case for the different PEDOT derivatives, proving that the previous change in dedoping dynamics is mainly due to the different voltages applied and not due to the structural differences between the films.

To facilitate the comparison of the PEDOT-crown derivatives in the three electrolytes, the dynamic curves were fit using a biexponential function, and the average time constant was calculated (Figures S23–S25). The trends for the different species (neutral ordered and disordered, singly charged, and doubly charged) are very similar (Figure S23). When dedoping to the same level (voltages indicated in Table S3), the time constants for the rise of disordered neutral species are comparable for PEDOT-C0 and -C25 in the three electrolytes (Figure 5c), and larger steps are needed to bring the systems to the same dedoping level. From PEDOT-C50 onward, the dedoping in the KPF_6_ electrolyte slows down significantly, as already discussed above, due to the decrease in driving force. The dedoping in the two electrolytes with Cl^–^ also slows down to some extent at higher crown ether content, but still more comparable time constants are observed. The slower dedoping dynamics for PF_6_^–^ in the polymers with high crown ether content (in spite of a higher voltage step compared to the other anions) is surprising, as we showed above that the nonhydrated PF_6_^–^ anion favors doping in the denser polymer structure and shifts the doping onset to more positive voltages. We hypothesize that this shift causes the slower dedoping, as we start at −0.4 V with a higher doping level than in the other electrolytes (Figure 4a). Moreover, the more favorable doping with PF_6_^–^ could point to stronger interactions with the polymer matrix that make the anion expulsion during dedoping more difficult. Unlike the dependence on the anion, the dedoping dynamics is independent of the noncompensating cation, without effects due to chelating with the crown ether (in contrast to findings of Wustoni et al.).^1,16^

Comparing the dynamics with the same voltage step of Δ*V* = 1.2 V, all PEDOT derivates in NaCl and KCl electrolytes exhibit almost the same time constant, and the slowing down of the dedoping above PEDOT-C50 is absent (Figure 5d). The dedoping time is also similar in the KPF_6_ electrolyte, but we still observe a weak slowing down from PEDOT-C50 onward. We relate the different behaviors with the PF_6_^–^ counterion again to the shifted redox window and different interactions taking place between this anion and polymer matrix, affecting the speed and necessary voltage at which this anion is expelled during the dedoping. It is indeed more difficult to dedope the films in the KPF_6_ electrolyte, as seen by the significant remaining doping level even at 0.8 V (Figure S9).

Finally, we also obtained the time constants for the doping process when switching the voltage back to the doped state at −0.4 V (Figure S23). The doping demonstrates overall much faster dynamics for all polymers, electrolytes, and investigated species, compared to the slower dedoping process. Therefore, it is impossible to detect any meaningful trends in the small time constants within experimental error.

### Film Thickness Dependence of the Dynamics

Different thicknesses of the PEDOT-crown films were investigated in the three electrolytes to further elucidate ion transport through increasing film volumes. Three thicknesses per PEDOT-crown derivative were measured, ranging from around 100 nm to around 400 nm (Table S1). SEM images for the different film thicknesses are depicted in Figure S3 and show that the thicker films of PEDOT-C0 have an even more fibrous microstructure with large pores compared to the thin ones, while the morphology of PEDOT-C100 (with small pores) is not affected by the electropolymerization time. For the different film thicknesses, the same MCR components were used, which means that the nanostructure of the polymers is insensitive to the thickness of the films. All spectroelectrochemistry data and their analysis are shown in the Supporting Information (SI).

We focus on the dedoping dynamics for the same voltage step of Δ*V* = 1.2 V (from −0.4 to 0.8 V). Especially for the thicker films, it is evident that in KCl and NaCl electrolytes, the dynamics are slower at less crown ether content and then get faster as more crown ether is present (Figure 6a). In the KPF_6_ electrolyte, the opposite trend is observed, and more crown ether content leads to a slowing down of the dedoping dynamics. In the thickest films of about 400 nm, a similar effect on the doping dynamics is observed (Figure S25). This is somewhat counterintuitive to the more favorable doping (with an onset at more positive voltage) seen with the PEDOT-C0 and PEDOT-C50 derivatives in all electrolytes, due to the larger pore size (Figure 4b). It indicates that the redox potential and dynamics might be ruled by different parameters, with the dynamics being more sensitive to interactions between the polymers and the anions. The crown ether side chains do not only change the ordering and porosity but also increase the polarity of the films, which favors interactions with more hydrated Cl^–^ ions and can lead to better ion transport and hence faster dynamics observed for PEDOT-C50, -C75, and -C100.^19,20^ In contrast, as PF_6_^–^ is hydrophobic and not significantly hydrated,^39^ its transport is faster in the hydrophobic PEDOT-C0 and -C25 polymers.

**Figure 6:**
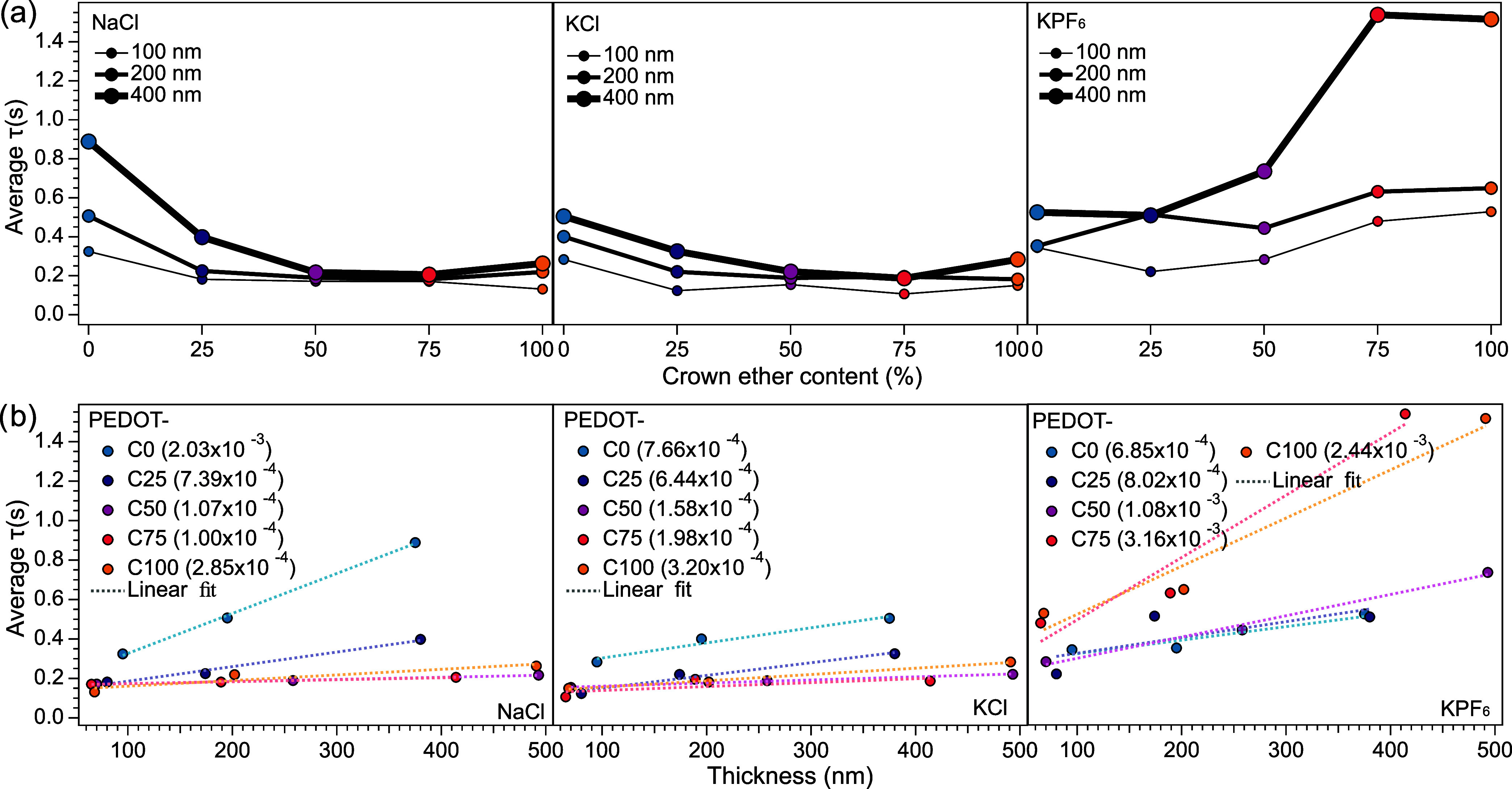
(a) Average dedoping time constant (for a voltage step from −0.4 to 0.8 V) for the PEDOT-crown derivatives with different film thicknesses of ∼100, 200, and 400 nm measured in the three electrolytes 0.1 M NaCl, KCl, and KPF_6_ (aq.). (b) Dependence of the average dedoping time constant on film thicknesses. The dashed lines show the linear fit for each PEDOT derivative, and the slope is indicated in the legend. The thickness dependence is shown for the three electrolytes 0.1 M NaCl, KCl, and KPF_6_ (aq.).

The impact of ion transport is exacerbated in the thicker films, where ions need to travel over longer distances. Interestingly, the thickness dependence of the dynamics differs significantly according to the crown ether content (Figure 6A). In the electrolytes with Cl^–^ anions, the dedoping time for PEDOT-C50 hardly slows down with film thickness, while there is a clear increase of the time constants for the PEDOT derivatives with either more or less crown ether content. The effect is further highlighted in Figure 6b, where the average dedoping time constant is plotted against the film thickness. The smaller the slope from the linear fit (indicated in the legend), the more independent the doping speed is of film thickness. For PEDOT-C50, the slope is one order of magnitude smaller in both NaCl and KCl compared to that of PEDOT-C0. For PEDOT-C75 and -C100, the slope increases again, but not to the same extent as in PEDOT-C0. In contrast, for KPF_6_, the slope is the largest for the polymers with high crown ether content, which have less affinity for the hydrophobic anion.

The thickness-independent ion transport in PEDOT-C50 is an important finding for OMIEC materials, where the switching speed is often limited by the time required for ions to diffuse through the polymer bulk. The nearly thickness-independent dynamics for PEDOT-C50 in chloride electrolytes indicates that ion transport remains efficient throughout the bulk of the film. This suggests that the microstructure of PEDOT-C50 provides continuous ionic transport pathways without bottlenecks. The behavior agrees with the higher volumetric capacitance of PEDOT-C50, which benefits from the best interplay of porosity, film order, and polarity to favor ionic transport and charge storage. While PEDOT-C0 develops a rough three-dimensional morphology with increasing film thickness (Figure S3), the crown-functionalized films grow more uniformly. However, the opposite extreme PEDOT-C100 forms a dense film structure, which is less favorable to ionic uptake. Thus, PEDOT-C50 in combination with Cl^–^ electrolytes represents an optimal compromise between structural order and ionic accessibility. In PEDOT-C50, the incorporation of crown ether moieties increases backbone ordering and film polarity, which improves compatibility with hydrates ions, while still maintaining sufficient porosity for ion permeation. In this system, upscaling to greater film thickness leads to higher volumetric capacitance without losing switching speed. This makes PEDOT-C50 in the chloride electrolyte a perfect candidate for applications in bioelectronics, biological sensing, and neuromorphic devices.^2,40^

## Conclusion

In this work, we built a modular polymer platform to systematically study how side-chain design and electrolyte choice affect the electrochemical doping of PEDOT derivatives. By the introduction of different ratios of 15-crown-5 moieties on the EDOT monomer repeat unit, we tuned the nanoscale ordering and porosity of the films and directly linked these structural changes to their electrochemical properties. With higher crown ether content, the neutral absorbance band becomes narrower and more structured, and SEM images show a smoother film with a reduced porosity. Spectroelectrochemistry combined with MCR analysis further shows that the insertion of crown ether shifts the balance of neutral ordered and disordered species, effectively extending the conjugation length of the charged species and narrowing the redox window widths due to the formation of more compact films with better chain ordering.

Comparing electrolytes shows that the polymer dictates the redox window width, whereas the redox onset is determined by the compensating anion of the electrolyte. In KPF_6_, the onset is shifted to more positive voltages compared to NaCl and KCl, indicating facilitated doping for the less hydrated anion. No measurable effect of the noncompensating cation (spectator ion) is observed despite the introduction of the chelating 15-crown-5 moieties.

From this systematic comparison, we identified PEDOT-C50 as the optimal PEDOT derivative in the series. It combines a favorable balance of nanoscale ordering, micrometer porosity, and film polarity, which results in the highest volumetric capacitance and the most efficient ion transport in the tested PEDOT-crown derivatives. In chloride-based electrolytes, PEDOT-C50 shows dedoping dynamics that are thickness-independent, which is unusual for OMIEC materials. This makes PEDOT-C50 a promising candidate for bioelectronic applications, where both capacitance and fast switching are critical.

## Supplementary Material



## Data Availability

The data that support the findings of this work are available as open access in the BORIS Repository of the University of Bern at DOI: 10.48620/98748.

## Notes

Additional details about samples and MCR analysis; additional GIWAXS, SEM, and EIS data; and additional results and analysis of the spectroelectrochemistry and doping/dedoping dynamics for different film thicknesses (PDF)
