# Supplementary material for: Structure-Driven Ion Transport in Conjugated Polymers with Crown Ether Side Chains: From the Nano- to Microscale
Source: Chem Mater. 2026 Jul 28;38(15):7614–25. doi: 10.1021/acs.chemmater.6c00382 (PMC13471735; doi:10.1021/acs.chemmater.6c00382)
Supplement: Supplementary file 1 [file cm6c00382_si_001.pdf]

Supplementary Information

Structure-driven ion transport in conjugated polymers with crown ether sidechains: From the nano- to microscale

Isabelle Heinzen[1], Ariel Lifer[2], Eyal Stein[2], Liad Hayoun[2], Christina J. Kousseff[3], Christian B. Nielsen[3], Gitti L. Frey[2], Natalie Banerji[1]*

[1]Department of Chemistry, Biochemistry and Pharmacy, University of Bern, Freiestrasse 3, 3012 Bern, Switzerland

[2]Department of Materials Science and Engineering, Technion – Israel Institute of Technology, Haifa, 32000, Israel

[3]Department of Chemistry, School of Physical and Chemical Sciences, Queen Mary University of London, Mile End Road, London E1 4NS, United Kingdom.

Table of Content

S1. Sample Overview 3
S2. GIWAXS Data 4
S3. Scanning electron microscopy images 5
3.1 Side-view SEM 5
3.2 Influence on polymerization time leading to thicknesses variation 6
S4. Multivariate curve resolution analysis 8
S5. Pore size distribution from the SEM images 11
S6. Electrochemical Impedance Spectroscopy (EIS) 13
S7. Spectroelectrochemistry 14
7.1 Spectra for ~100 nm films 14
7.2 Spectra for ~200 nm films 16
7.3 Spectra for ~400 nm films 19
S8. Comparison of ordered and disordered neutral areas 22
8.1 Dedoped films (at 0.8 V) 22
8.2 Doped films 23
8.3 Ratio of neutral species in the dedoped films 24
S9. Shift in absorbance maximum of singly charged species 25
S10. Species evolution with applied voltage 26
10.1 For ~100 nm films 26
10.2 For ~200 nm films 27
10.3. For ~400 nm films 28
S.11 Comparable doping level 29
S12. Doping and dedoping dynamics 30
12.1 Time constants for ~100 nm films 32
12.2 Time constants for ~200 nm films 33
12.3 Time constants for ~400 nm films 34
S13. References 35

S1. Sample Overview

Material	Thin film (~100 nm)	Medium film (~200 nm)	Thick film (~400 nm)
PEDOT-C0	95 nm	195 nm	375 nm
PEDOT-C25	80 nm	174 nm	380 nm
PEDOT-C50	70 nm	258 nm	493 nm
PEDOT-C75	65 nm	189 nm	414 nm
PEDOT-C100	68 nm	202 nm	491 nm

Table S1. Sample overview of the measured PEDOT-crown derivatives and their corresponding thickness measured with white-light interferometry.

S2. GIWAXS Data

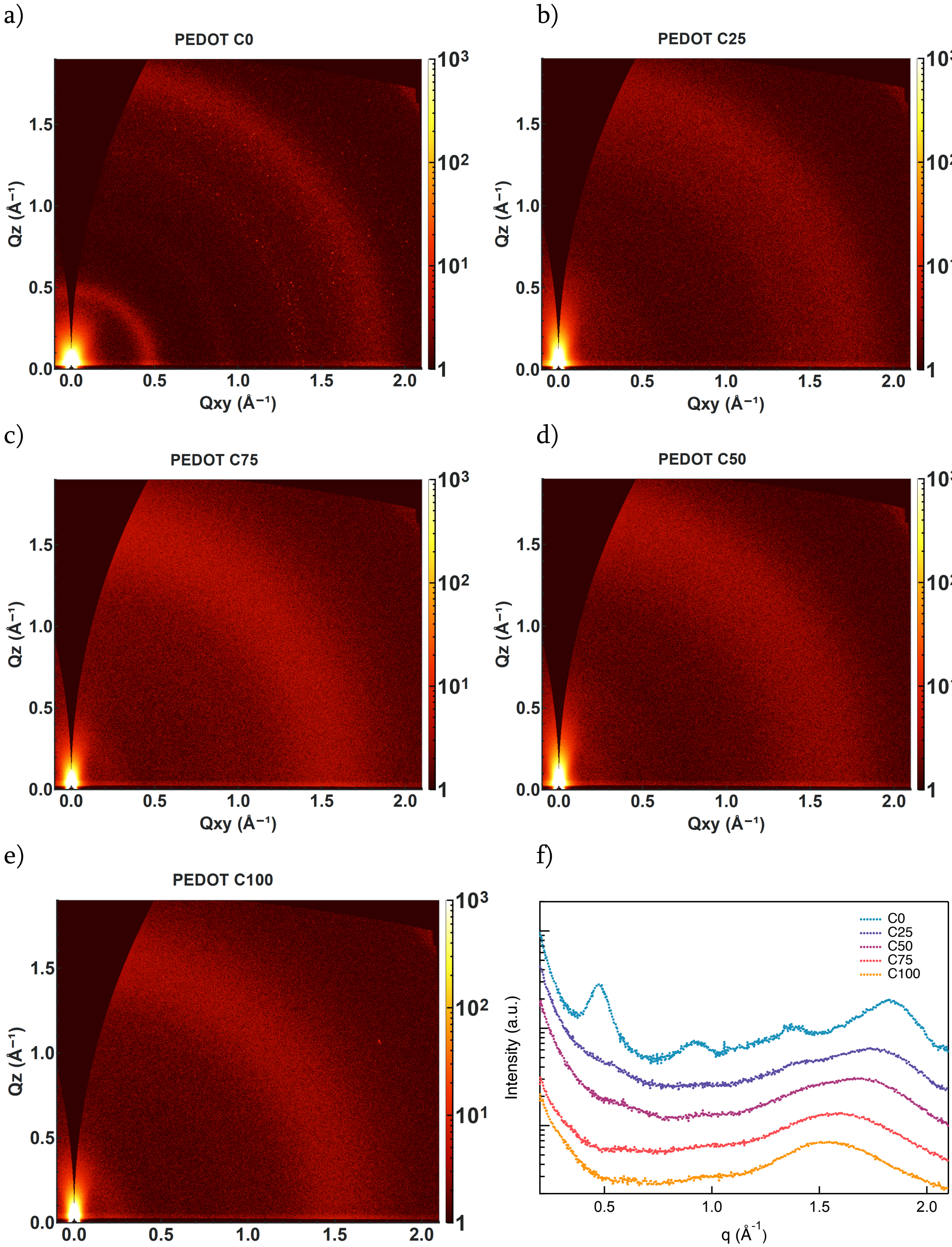

Figure S1. a)-e) GIWAXS 2D diffractograms and f) linecuts integrated from 0 to 90° for the PEDOT-crown series.

S3. Scanning electron microscopy images

3.1 Side-view SEM

The scanning electron microscopy (SEM) measurements taken from the side view support the finding from the top view images. The PEDOT-crown polymers with more crown ether showcase a smoother film surface and are less porous.

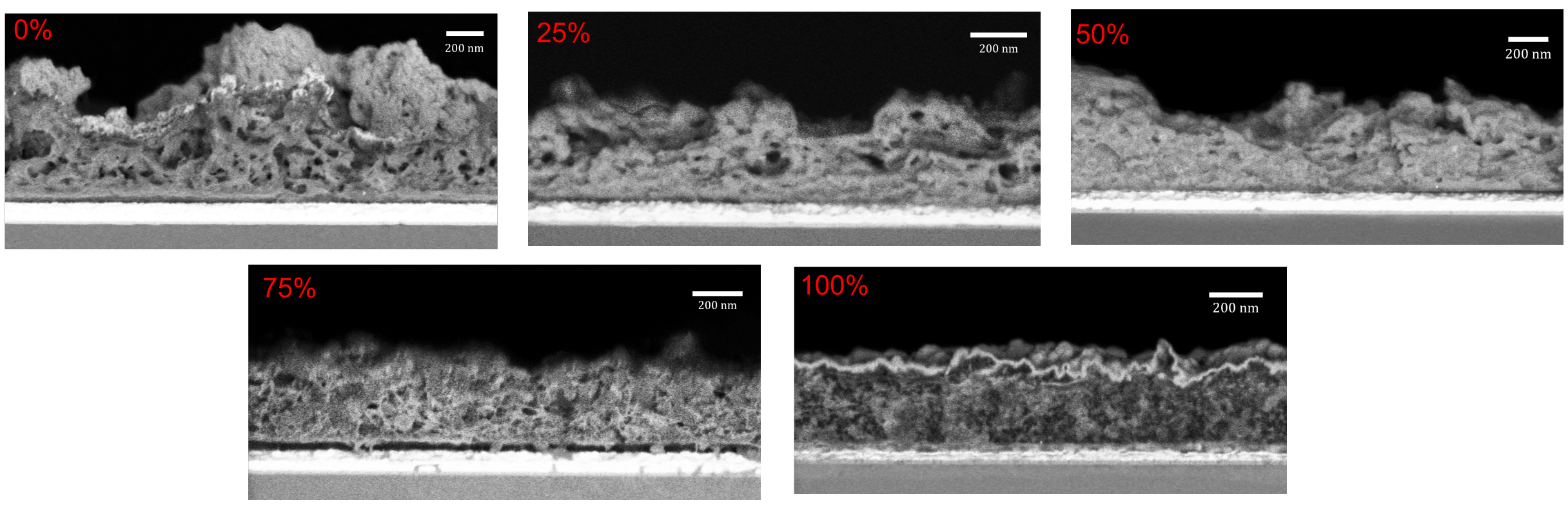

Figure S2. SEM images of the PEDOT-crown derivates showing the porosity and degree of smoothness from the side view. To enhance morphological contrast, vapor phase infiltration (VPI) was applied prior to imaging using sequential pulses of diethylzinc (DEZ) and water as precursors. The VPI-treated samples were then prepared for cross-sectional imaging by cryo-fracturing, which involved cooling in liquid nitrogen and cleaving in a custom three-point bending apparatus. The images were acquired using an in-lens energy-selective backscattered (EsB) detector with a grid voltage of 490 V.

3.2 Influence on polymerization time leading to thicknesses variation

The electropolymerization time can control the film thickness and influences the morphology. Figure S2 shows top-view SEM images of PEDOT-C0 and PEDOT-C100 films grown to ~150 nm and ~450 nm thickness, which were prepared using two different polymerization durations. These films were not VPI treated prior to the SEM characterization. Unlike the VPI-treated sample these films display smooth surfaces. For PEDOT-C0, morphology depends strongly on the polymerization time. Thicker films (~450 nm) form an interconnected network structure. Thinner films (~150 nm) appear more connected and less open, with numerous small pores across the surface, suggesting that the fibrous morphology develops progressively with polymerization time. PEDOT-C100 films display similar morphology at both thicknesses, indicating that their structural development is less sensitive to polymerization time within the range tested.

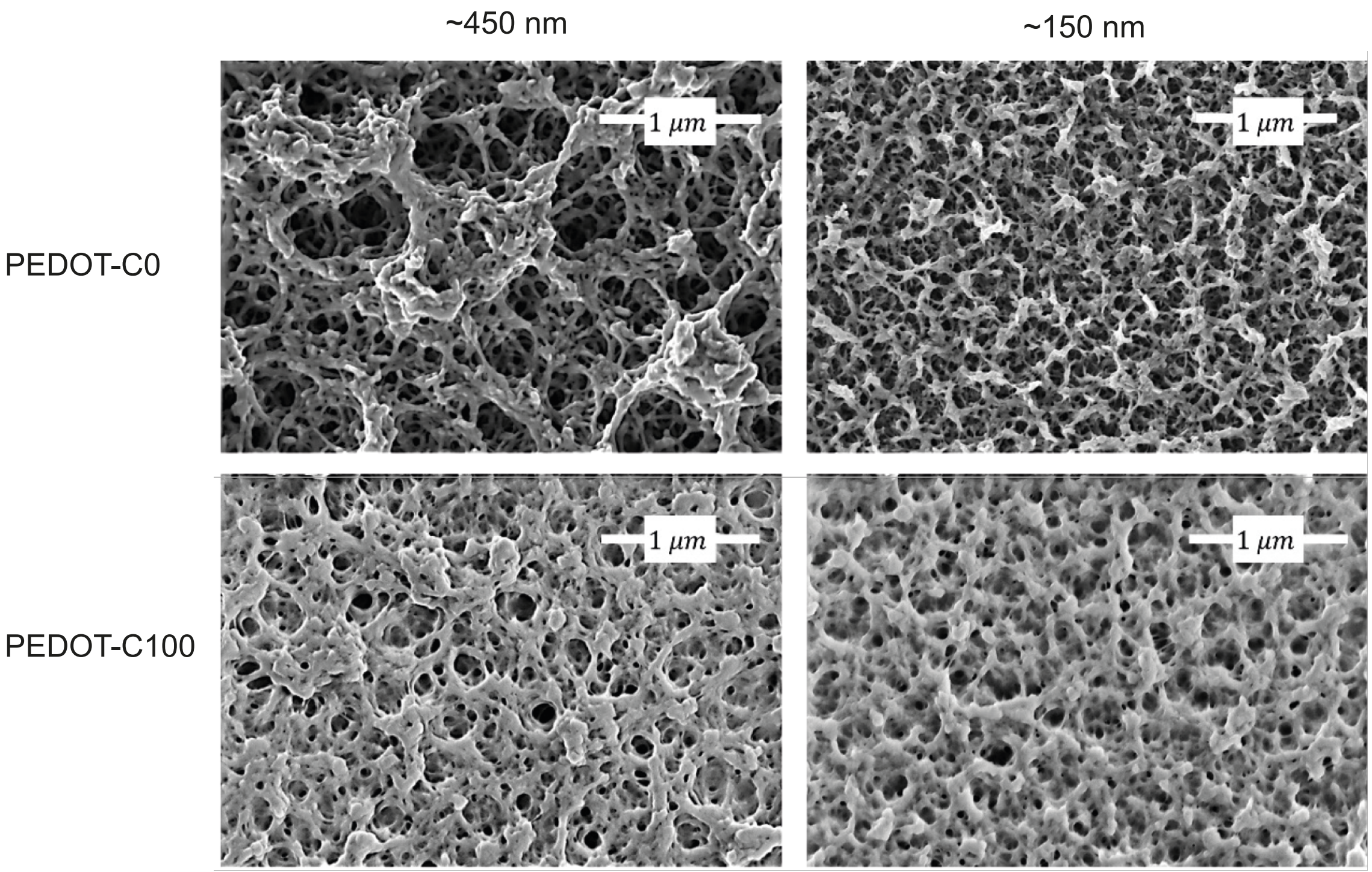

Figure S3: Top-view SEM images comparing the morphology of PEDOT-C0 (top row) and PEDOT-C100 (bottom row) films. Films were prepared with different polymerization times, corresponding to target thicknesses of approximately 150 nm and 450 nm based on prior calibration.

S4. Multivariate curve resolution analysis

Multivariate curve resolution (MCR) is a chemometric technique used to analyze mixture data by separating the relative abundances and the signatures of the pure analytes. We employed MCR to spectroscopy to resolve spectra and concentration of various species (neutral ordered, neutral disordered, singly charged and doubly charges species) from the in-situ time-resolved Vis-NIR spectra. The analysis is based on the following formula:

$$D = CS^T + \varepsilon$$

Here, D denotes the spectral dataset, while MCR determines the solution through alternating regression. In this expression, C corresponds to the concentration, S to the spectral signatures and epsilon to the residual, including noise and errors into the model. During the alternating regression the C is fixed while performing multivariate multiple regression for S and *vice versa*, this continues until the best possible estimation is accomplished.[1]

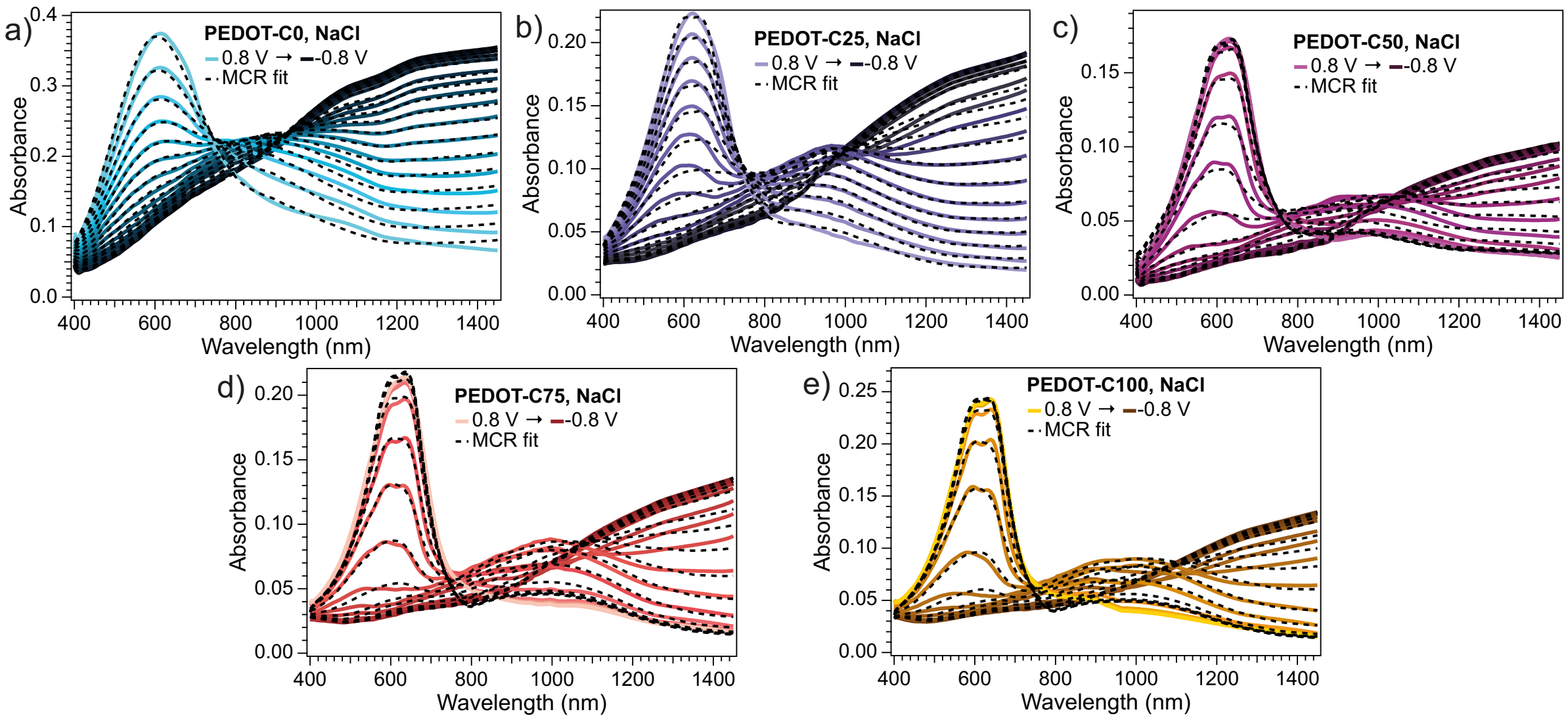

Figure S4. Steady-state absorbance spectra for ~100 nm films in 0.1 M NaCl upon the application of doping voltages ranging from 0.8 to -0.8 V (ΔV = 0.1 V) for a) PEDOT-C0, b) PEDOT-C25, c) PEDOT-C50, d) PEDOT-C75, e) PEDOT-C100 and. The fit from the MCR is depicted with dashes lines in the spectra a)-e).

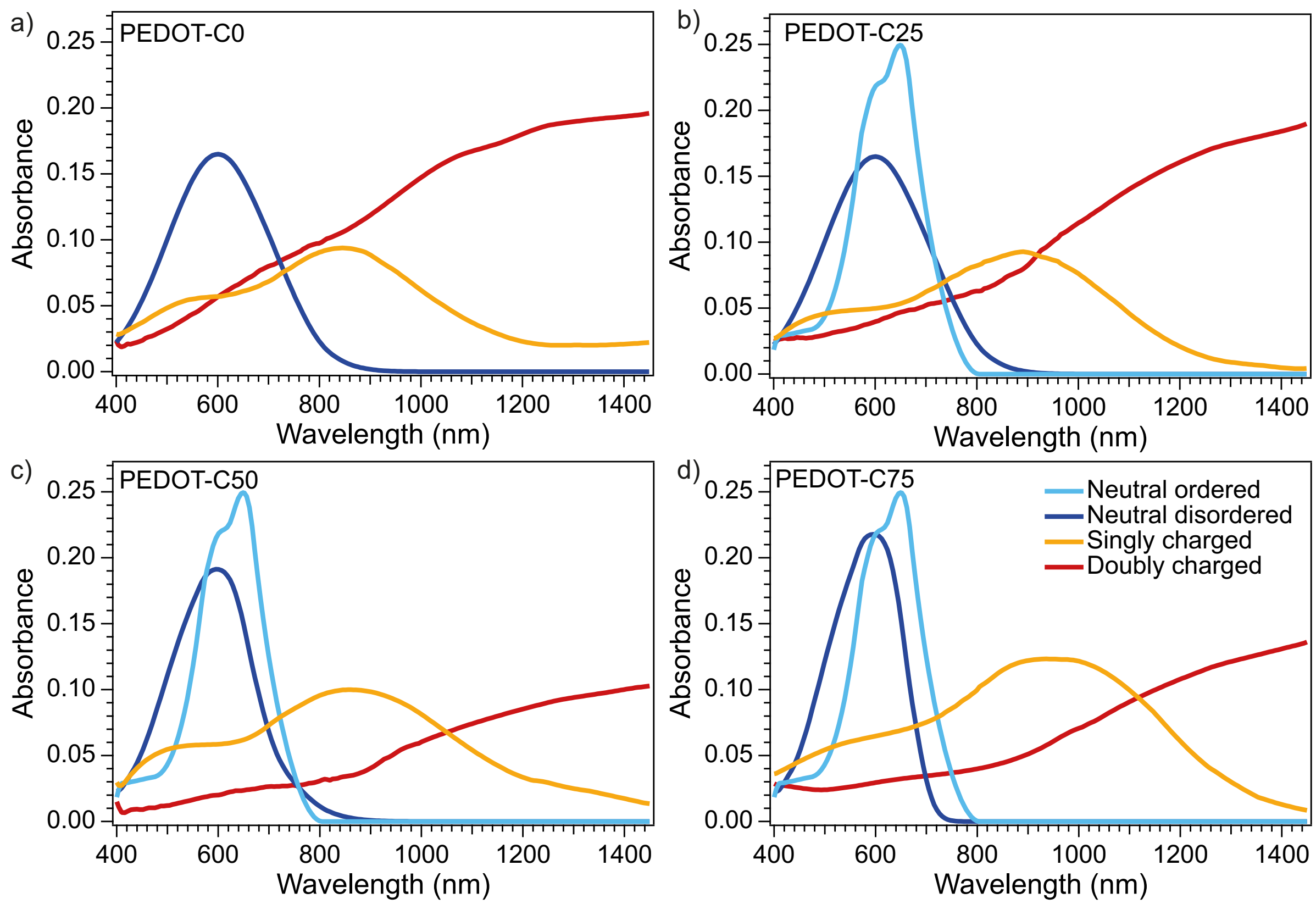

Figure S5. Spectral signatures obtained by MCR analysis of a) PEDOT-C0, b) PEDOT-C25, c) PEDOT-C50 and d) PEDOT-C75.

S5. Pore size distribution from the SEM images

To quantify the morphological changes in the PEDOT-crown derivatives, the top-view SEM images were segmented into binary representations by applying a brightness threshold over the original grey scale SEM images. Pixels were classified as either pores (voids) or polymer. **The grayscale threshold was selected individually for each HRSEM image based on visual identification of pore regions, due to variations in imaging conditions. While the absolute pore area fraction depends on the threshold choice, the observed trends were robust to reasonable variations in this parameter**. A representative image from the PEDOT-C0 film is in Figure S6 a). Feature analysis of these binary maps provided pore statistics (Figure S6 b)). Since this approach relies on grayscale intensity-based thresholding of 2D SEM micrographs, it offers an estimate rather than exact values but captures clear trends. The pore area percentage decreases linearly with increasing EDOT-Crown content.

For the quantification of the pore size distribution for the different amount of crown ether the area was calculated based on the area weight.

$$\overline{D} = \sum_{i=1}^{N} D_i \cdot w_i$$

Where $\overline{D}$ is the average pore size, D_i is the size of the pore i and w_i is the area-based weight of the pore, which is calculated by the following formula:

$$w_i = \frac{D_i}{Total\ Pores\ Area}$$

It is observed that with an increase of the crown percentage, the area averaged pore size decreases, indicating that large pores are progressively suppressed and become smaller with increasing crown ether content.

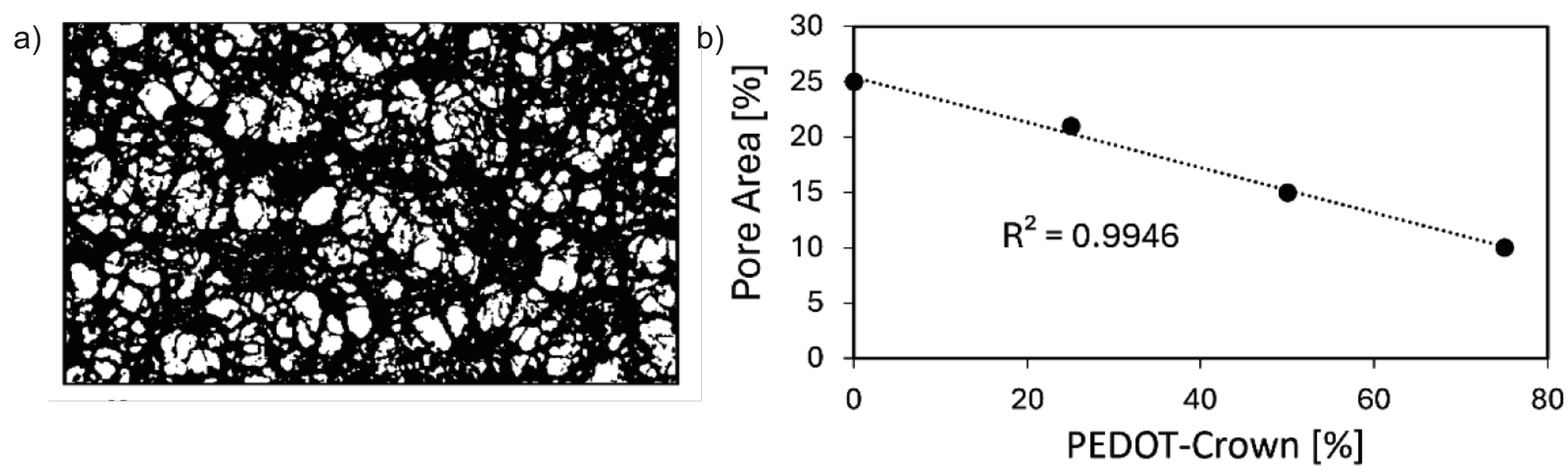

Figure S6: Quantitative analysis of morphological features from top-view SEM. a) Representative binary-processed SEM image of a PEDOT-C0 film used for feature segmentation. b) Percentage of total image area occupied by pores as a function of EDOT-Crown monomer percentage.

S6. Electrochemical Impedance Spectroscopy (EIS)

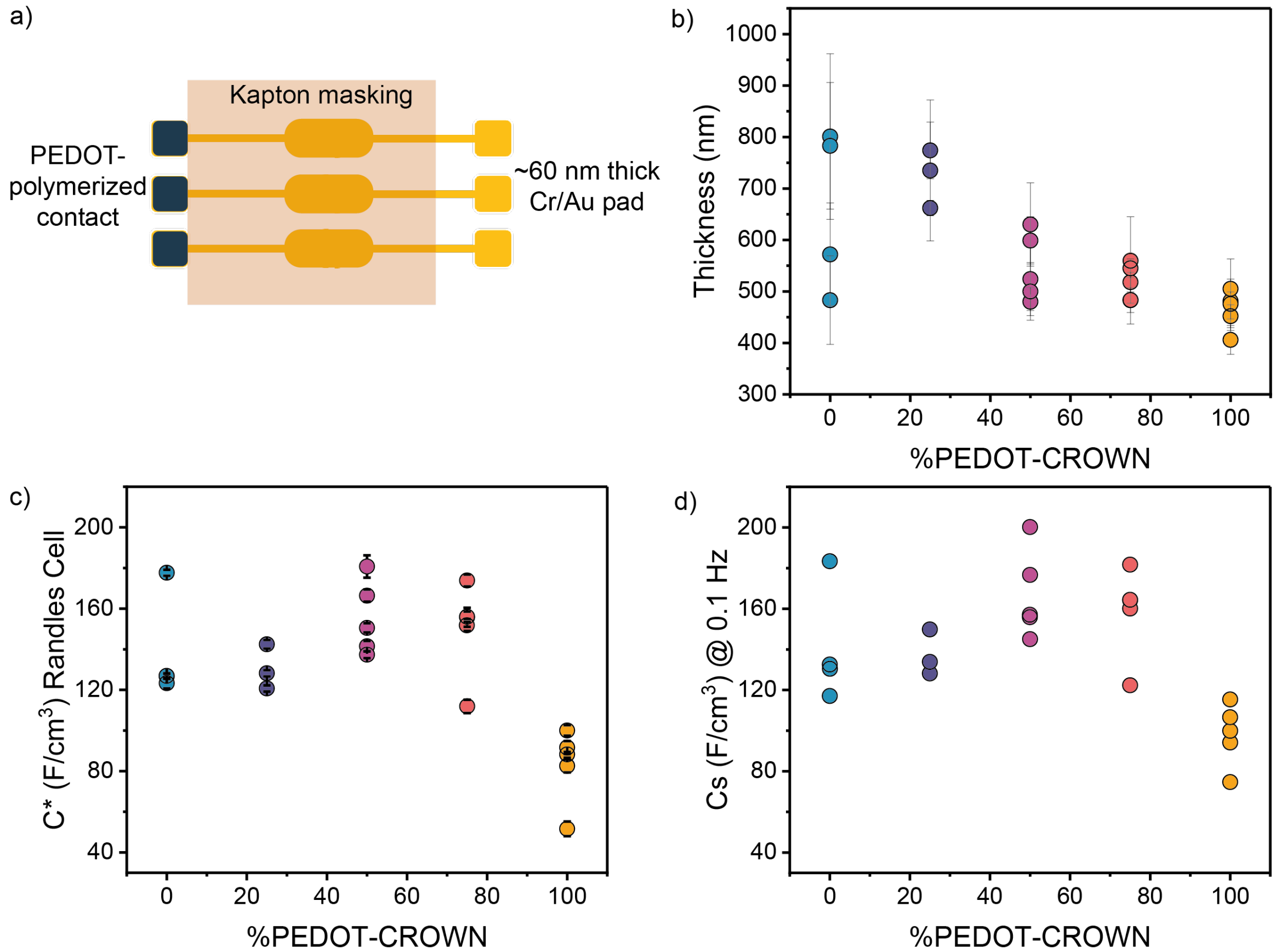

Figure S7. a) Scheme of the EIS measurement gold pads. b) Thicknesses of the measured PEDOT-crown films, the thickness was measured using a mechanical profilometer. The capacitance was measured at 0.1 Hz and 0 V (vs. Ag/AgCl) using a 0.1 M NaCl electrolyte. Two methods were applied to for the fitting c) Randles Cell R (RC) and d) Series capacitance. For both fittings one parameter was excluded from the results, marked in the graphs as grey/red marker.

S7. Spectroelectrochemistry

7.1 Spectra for ~100 nm films

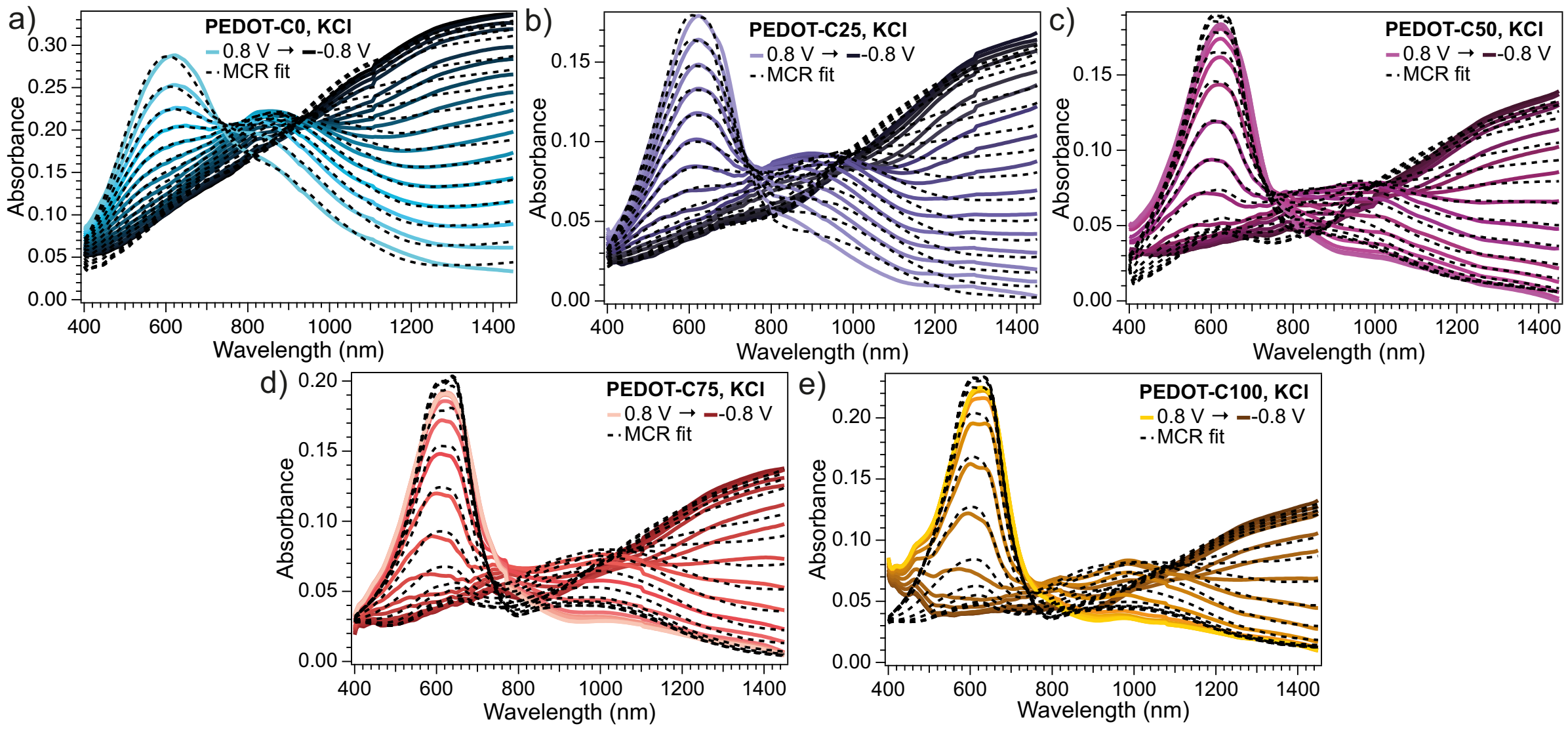

Figure S8. Steady-state absorbance spectra for ~100 nm films in 0.1 M KCl upon the application of doping voltages ranging from 0.8 to -0.8 V (ΔV = 0.1 V) for a) PEDOT-C0, b) PEDOT-C25, c) PEDOT-C50, d) PEDOT-C75, e) PEDOT-C100 and. The fit from the MCR is depicted with dashes lines in the spectra a)-e).

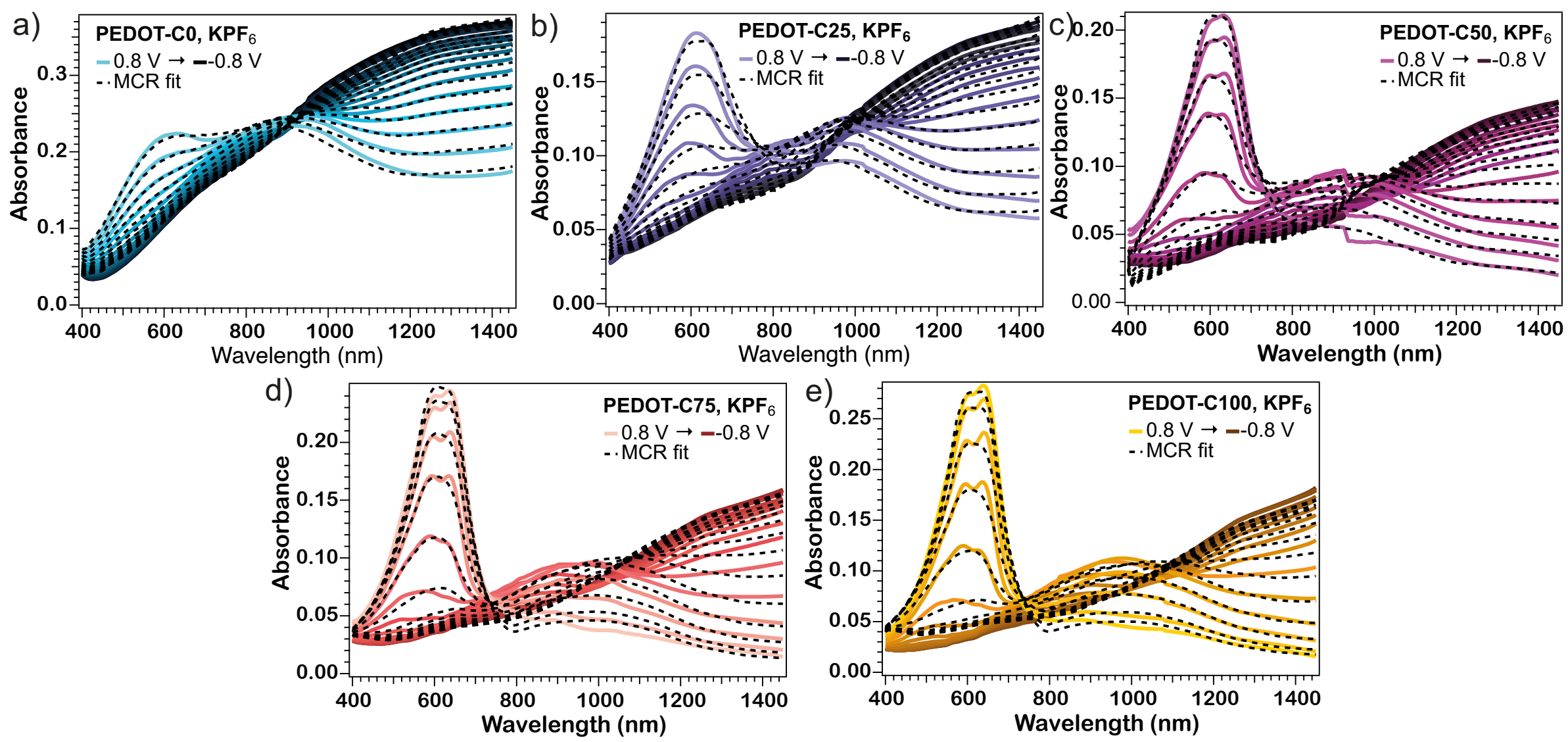

Figure S9. Steady-state absorbance spectra for ~100 nm films in 0.1 M KPF_6 upon the application of doping voltages ranging from 0.8 to -0.8 V (ΔV = 0.1 V) for a) PEDOT-C0, b) PEDOT-C25, c) PEDOT-C50, d) PEDOT-C75, e) PEDOT-C100 and. The fit from the MCR is depicted with dashes lines in the spectra a)-e).

7.2 Spectra for ~200 nm films

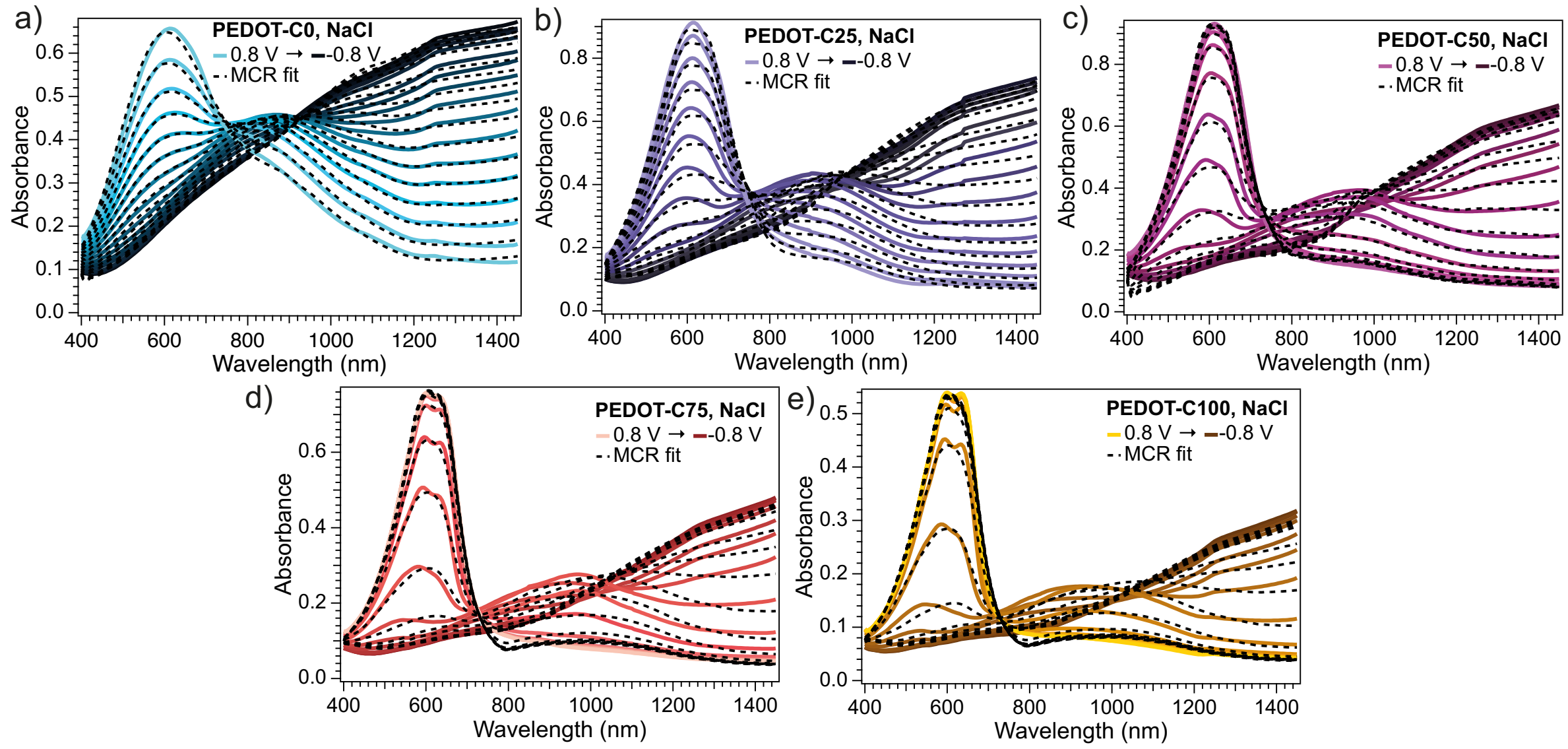

Figure S10. Steady-state absorbance spectra for ~200 nm films in 0.1 M NaCl upon the application of doping voltages ranging from 0.8 to -0.8 V (ΔV = 0.1 V) for a) PEDOT-C0, b) PEDOT-C25, c) PEDOT-C50, d) PEDOT-C75, e) PEDOT-C100 and. The fit from the MCR is depicted with dashes lines in the spectra a)-e).

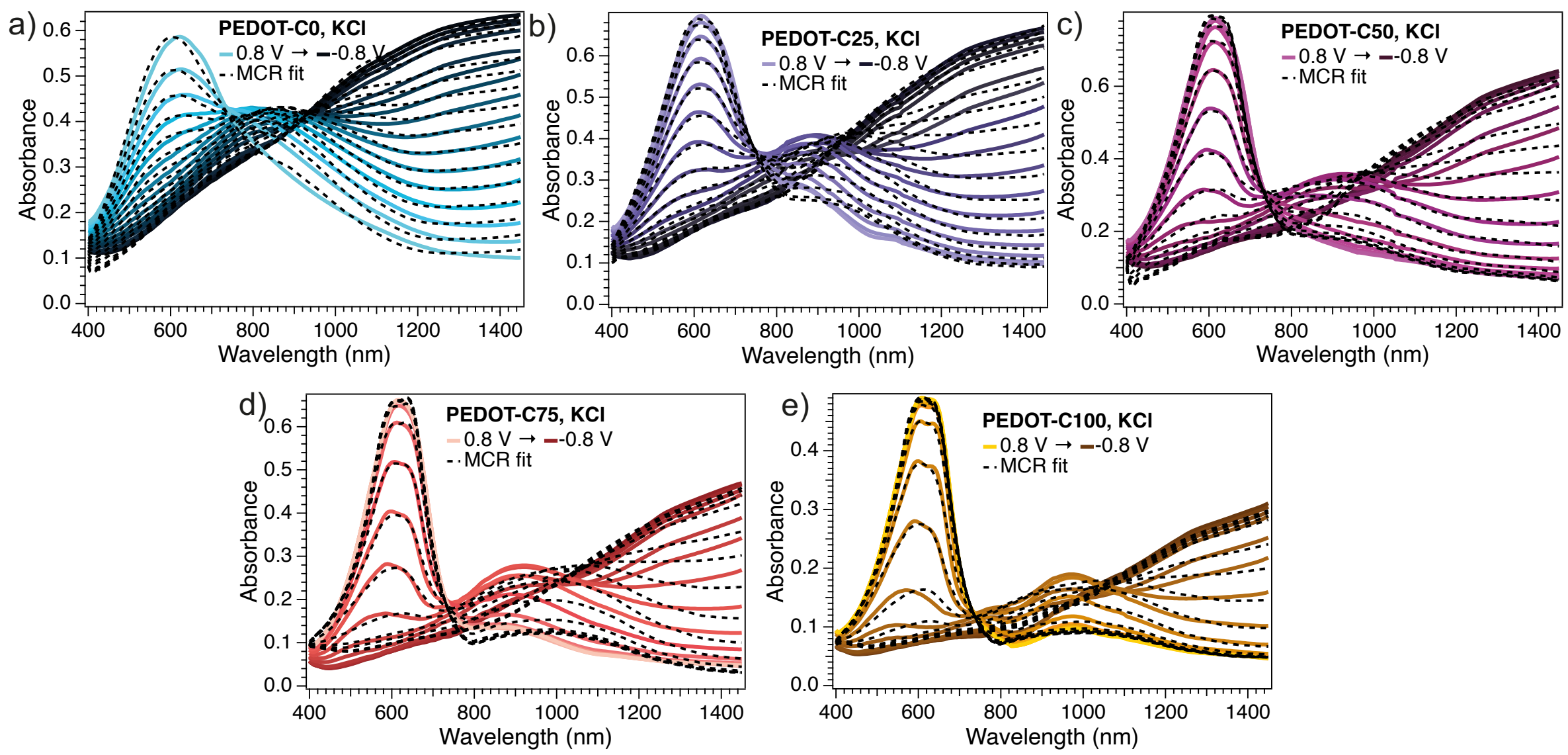

Figure S11. Steady-state absorbance spectra for ~200 nm films in 0.1 M KCl upon the application of doping voltages ranging from 0.8 to -0.8 V (ΔV = 0.1 V) for a) PEDOT-C0, b) PEDOT-C25, c) PEDOT-C50, d) PEDOT-C75, e) PEDOT-C100 and. The fit from the MCR is depicted with dashes lines in the spectra a)-e).

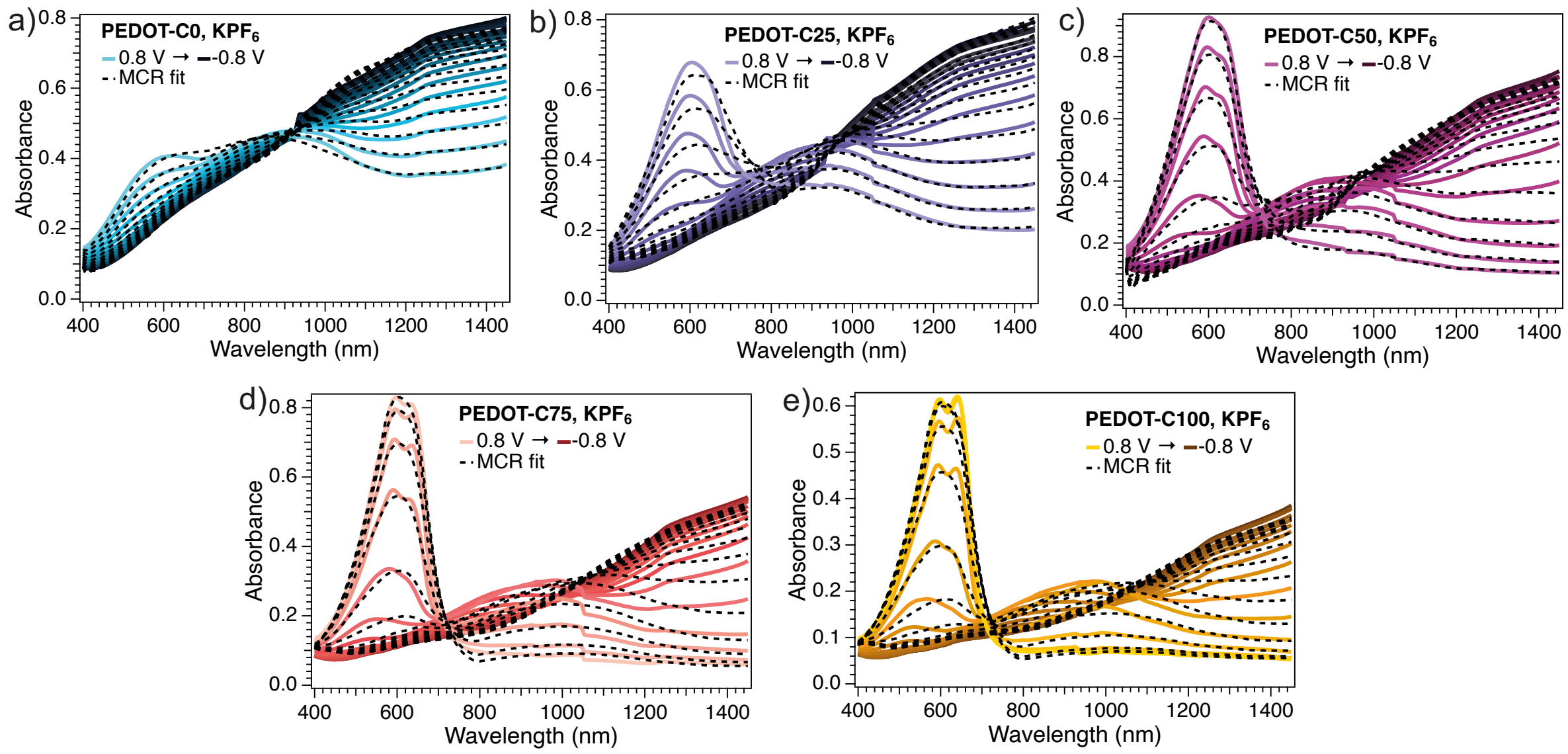

Figure S12. Steady-state absorbance spectra for ~200 nm films in 0.1 M KPF_6 upon the application of doping voltages ranging from 0.8 to -0.8 V (ΔV = 0.1 V) for a) PEDOT-C0, b) PEDOT-C25, c) PEDOT-C50, d) PEDOT-C75, e) PEDOT-C100 and. The fit from the MCR is depicted with dashes lines in the spectra a)-e).

7.3 Spectra for ~400 nm films

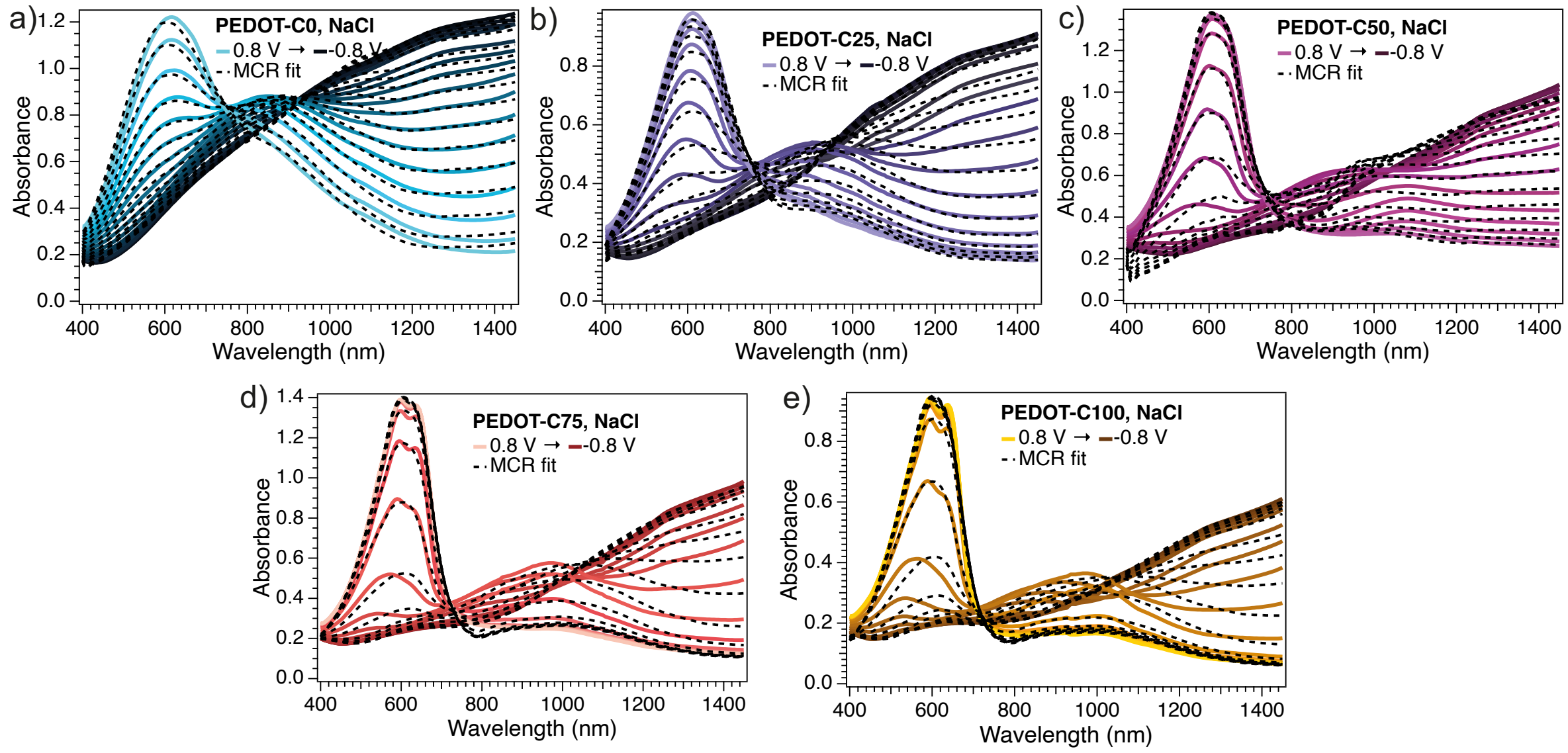

Figure S13. Steady-state absorbance spectra for ~400 nm films in 0.1 M NaCl upon the application of doping voltages ranging from 0.8 to -0.8 V (ΔV = 0.1 V) for a) PEDOT-C0, b) PEDOT-C25, c) PEDOT-C50, d) PEDOT-C75, e) PEDOT-C100 and. The fit from the MCR is depicted with dashes lines in the spectra a)-e).

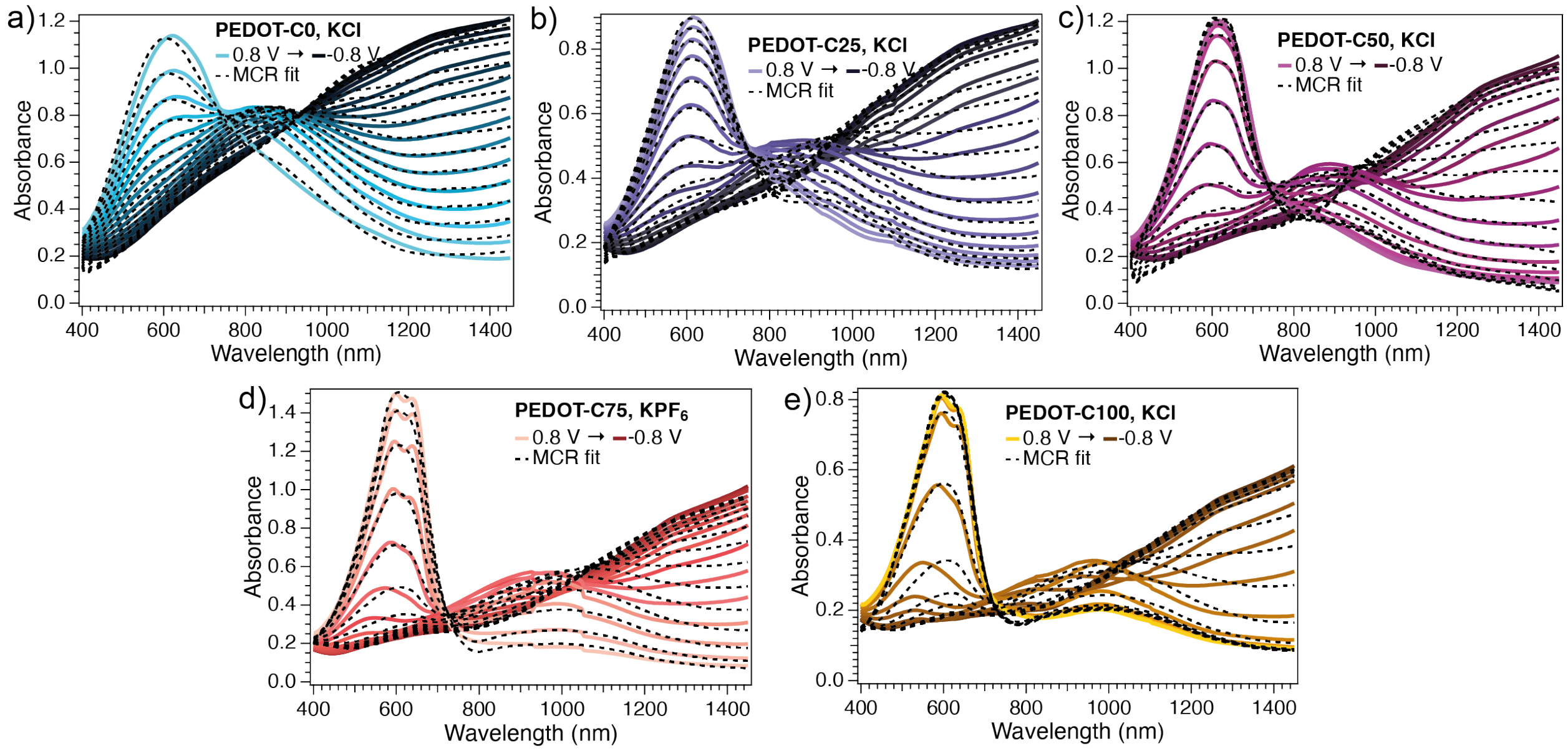

Figure S14. Steady-state absorbance spectra for ˜400 nm films in 0.1 M KCl upon the application of doping voltages ranging from 0.8 to -0.8 V (ΔV = 0.1 V) for a) PEDOT-C0, b) PEDOT-C25, c) PEDOT-C50, d) PEDOT-C75, e) PEDOT-C100 and. The fit from the MCR is depicted with dashes lines in the spectra a)-e).

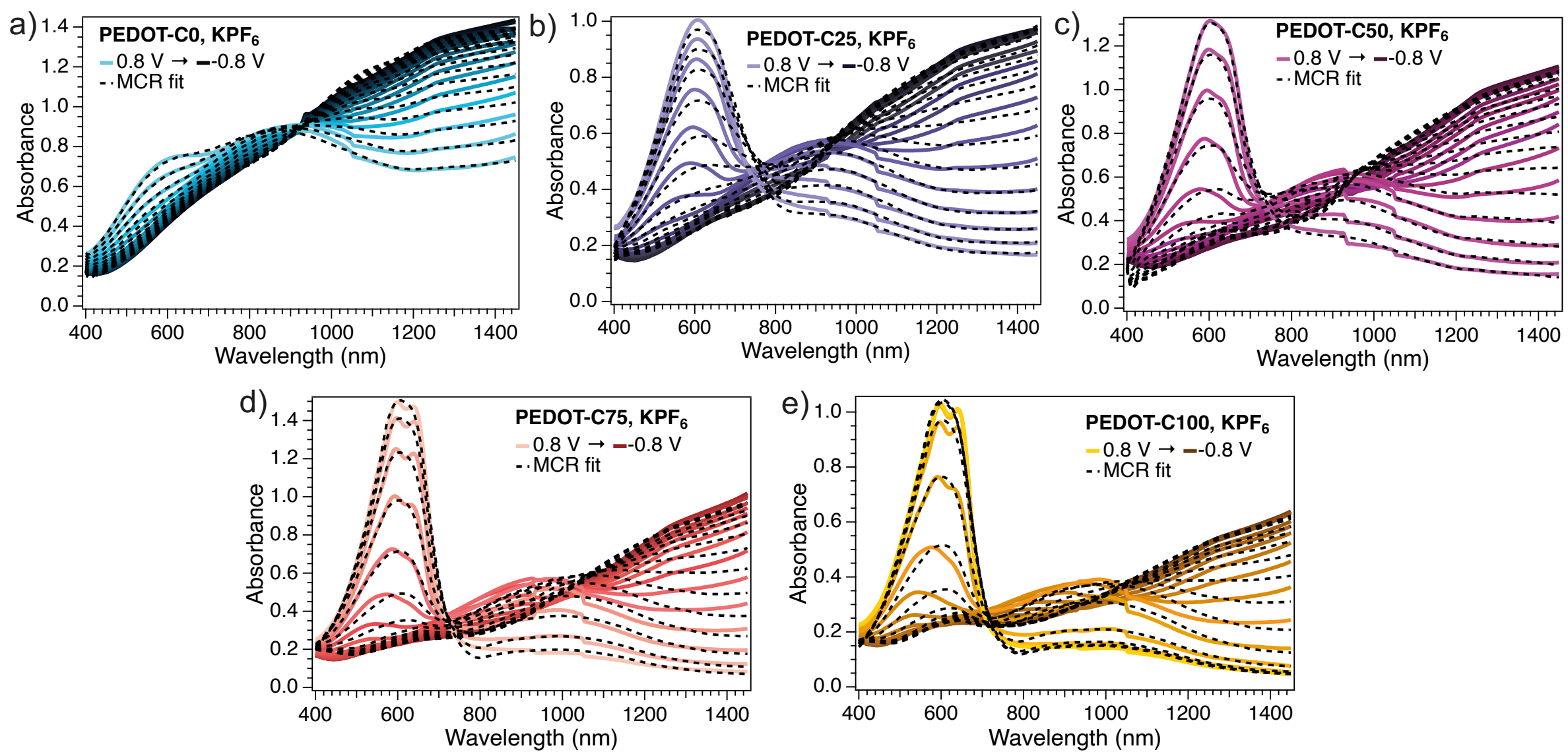

Figure S15. Steady-state absorbance spectra for ~400 nm films in 0.1 M KPF_6 upon the application of doping voltages ranging from 0.8 to -0.8 V (ΔV = 0.1 V) for a) PEDOT-C0, b) PEDOT-C25, c) PEDOT-C50, d) PEDOT-C75, e) PEDOT-C100 and. The fit from the MCR is depicted with dashes lines in the spectra a)-e).

S8. Comparison of ordered and disordered neutral areas

8.1 Dedoped films (at 0.8 V)

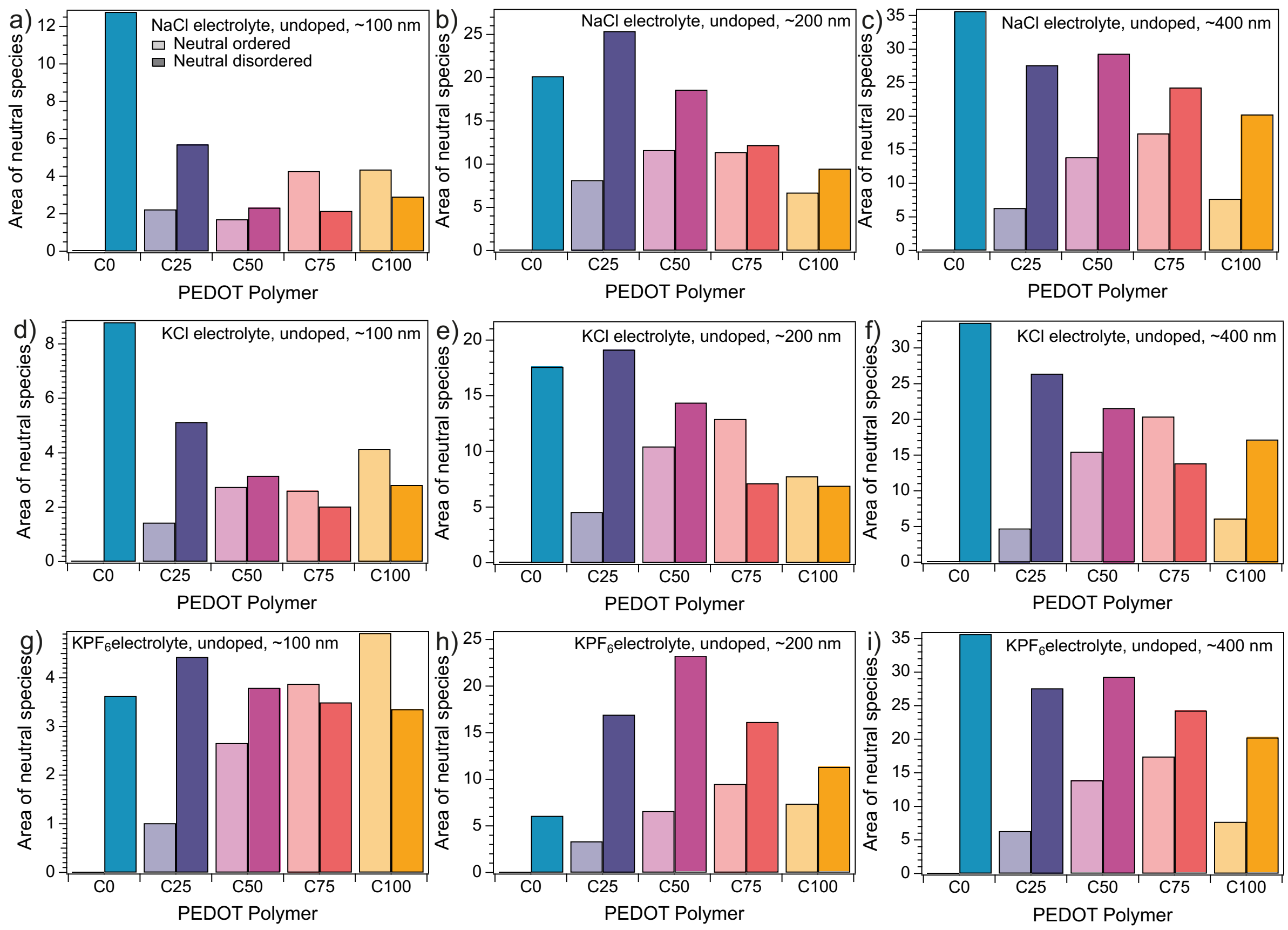

Figure S16. Area of the deconvoluted spectral bands corresponding to ordered and disordered neutral species from MCR analysis in the dedoped films (0.8 V) for 0.1 M NaCl (a-c), 0.1 M KCl (d-f) and 0.1 M KPF_6 (g-i). The same trend can be observed over all the thicknesses, which are depicted in the graph: ~100 nm (a, d, g), ~200 nm (b, e, h) and ~400 nm (c, f, i). There are some deviations in KPF_6 compared to the other electrolytes. The neutral disordered species seems to be less abundant compared to NaCl and KCl electrolyte. However, this is an effect due to the incomplete dedoping of PEDOT-C0 in KPF_6 electrolyte.

8.2 Doped films

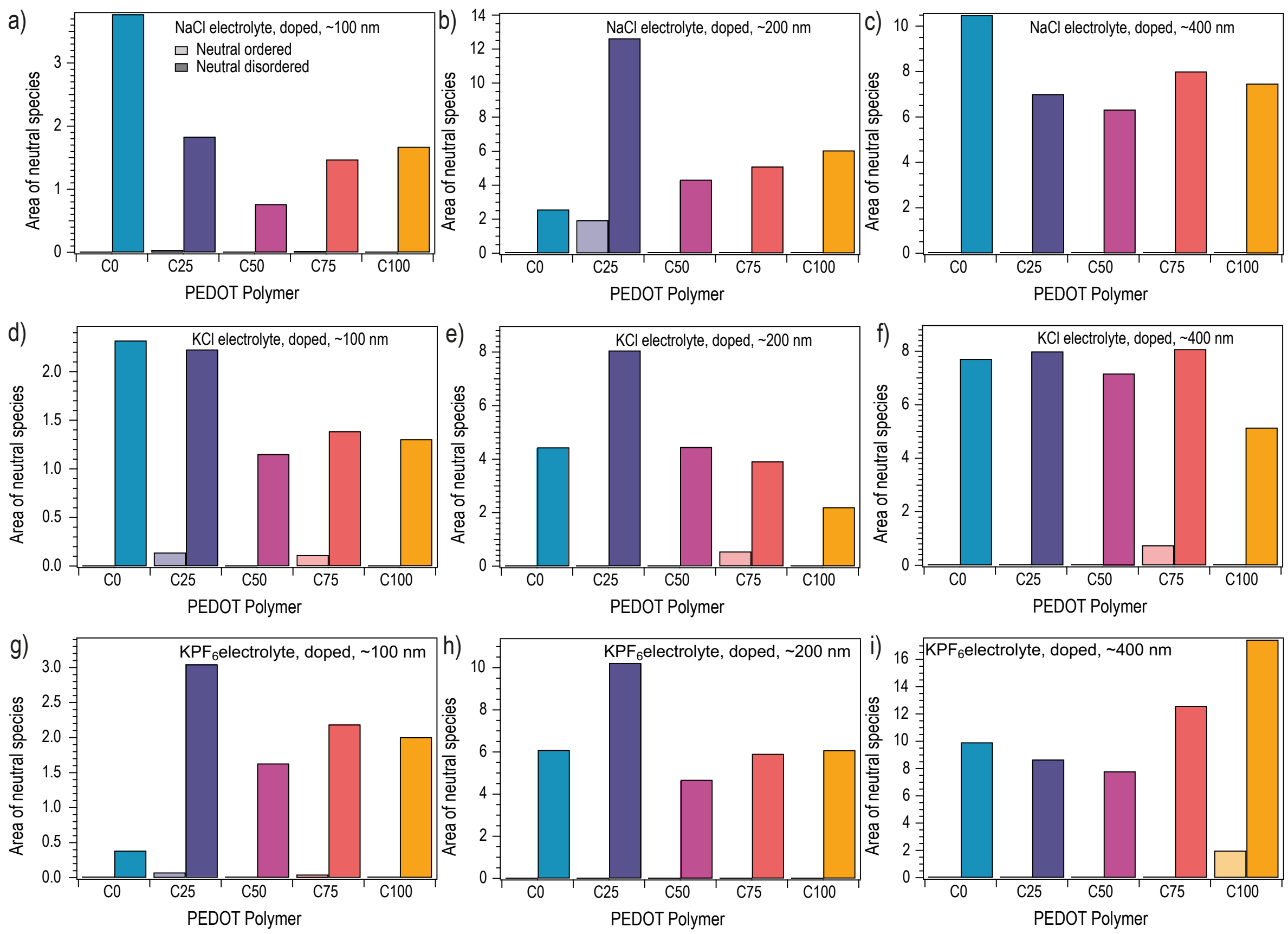

Figure S17. Area of the deconvoluted spectral bands corresponding to ordered and disordered neutral species from MCR analysis in the doped films (at a voltage, where the maximum absorbance of neutral/singly charges species = 1), for 0.1 M NaCl (a-c), 0.1 M KCl (d-f) and 0.1 M KPF_6 (g-i). The same trend can be observed over all the thicknesses, which are depicted in the graph: ~100 nm (a, d, g), ~200 nm (b, e, h) and ~400 nm (c, f, i).

8.3 Ratio of neutral species in the dedoped films

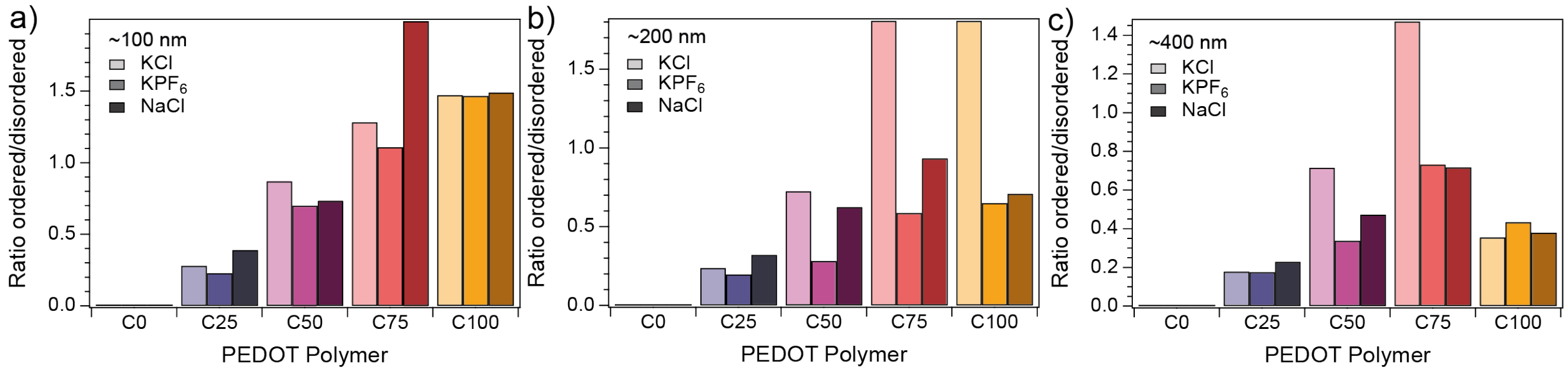

Figure S18. Ratio of the neutral ordered and disordered species area in the dedoped films (0.8 V) for a) ~100 nm, b) ~200 nm and c) ~400 nm film thickness. The different electrolytes are depicted as different shading.

S9. Shift in absorbance maximum of singly charged species

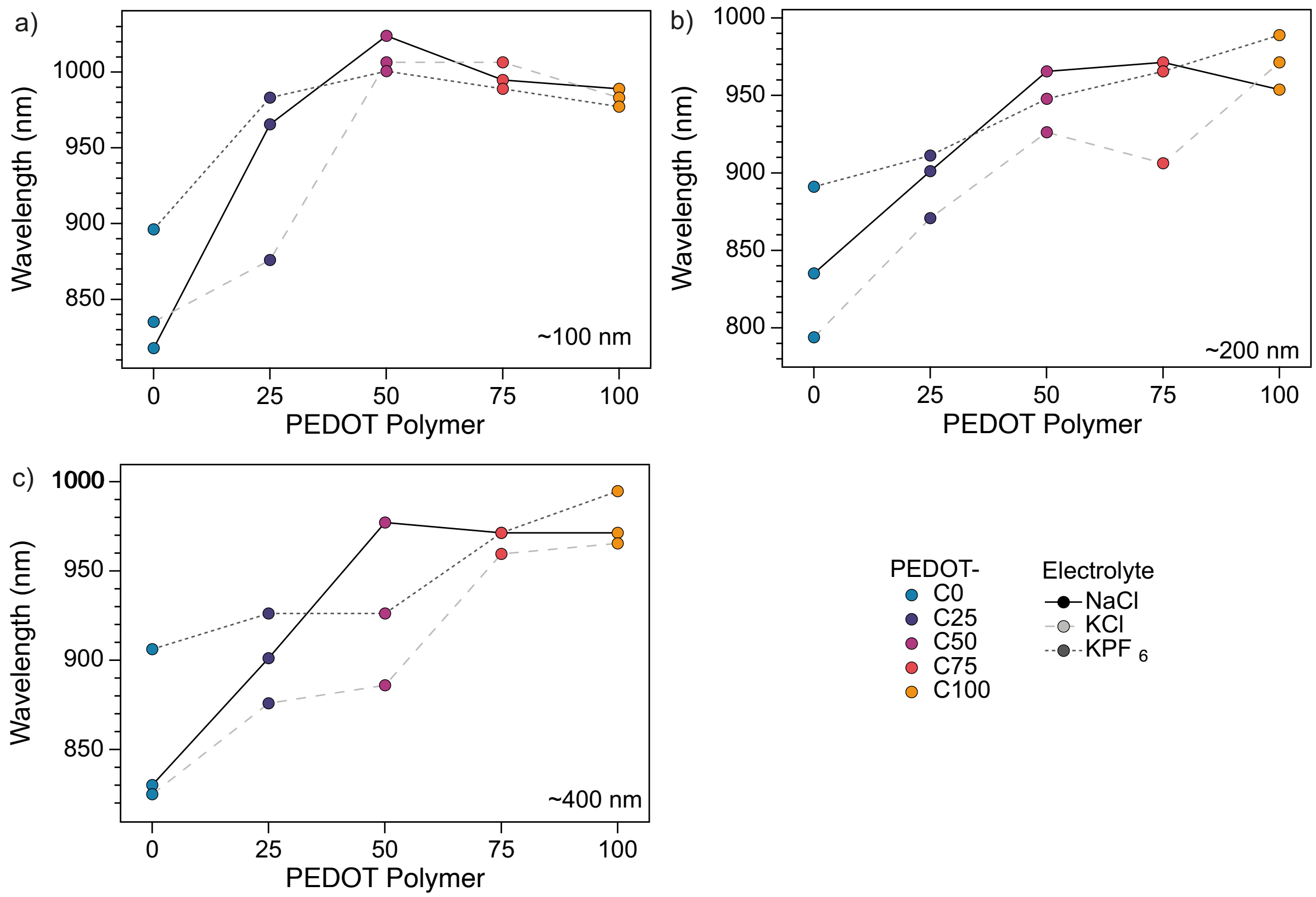

Figure S19. Shift of the singly charged species absorbance maximum for doped PEDOT-crown polymer with a thickness of a) ~100 nm, b) ~200 nm and c) ~400 nm.

S10. Species evolution with applied voltage

10.1 For ~100 nm films

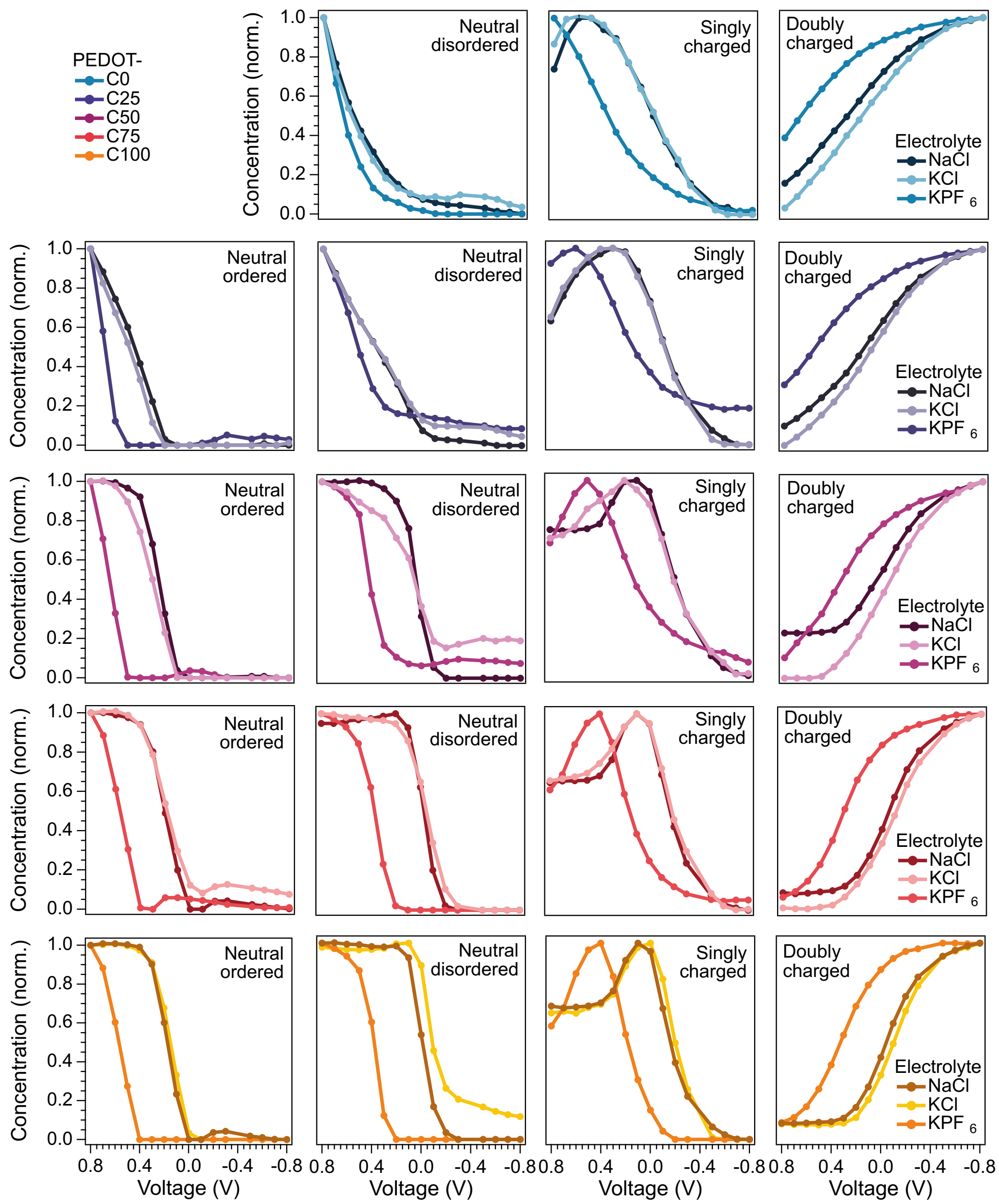

Figure S20. Species concentrations of the ~100 nm films as a function of voltage (first column: neutral ordered, second column: neutral disordered, third column: singly charged species, fourth column: doubly charged species), and the different crown polymers (first row: PEDOT-C0, second row: PEDOT-C25, third row: C50, fourth row: C75, fifth row: C100). The same measurements were carried out in 0.1 M NaCl, 0.1 M KCl and 0.1 M KPF_6.

10.2 For ~200 nm films

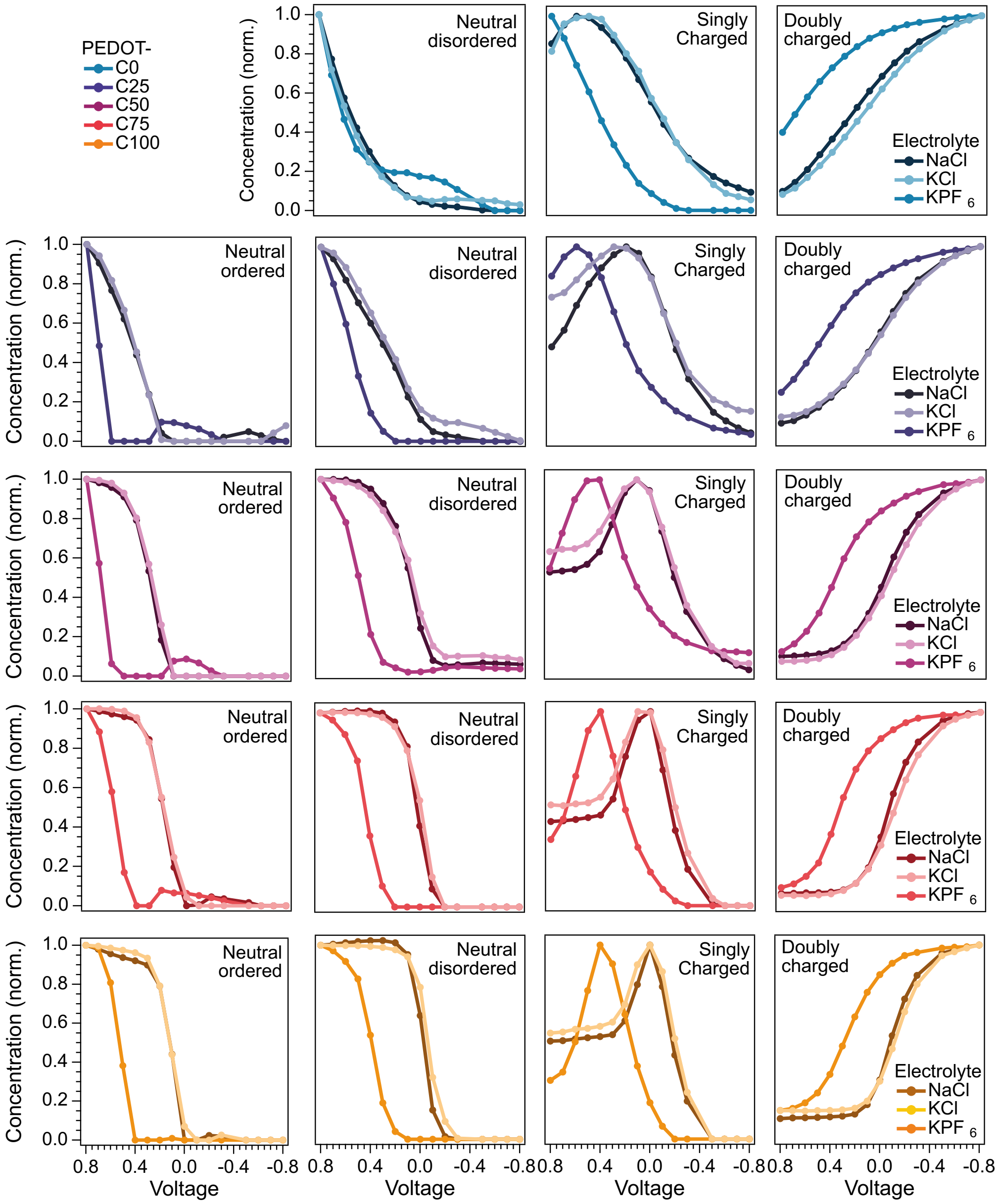

Figure S21. Species concentrations of the ~200 nm films as a function of voltage (first column: neutral ordered, second column: neutral disordered, third column: singly charged species, fourth column: doubly charged species), and the different crown polymers (first row: PEDOT-C0, second row: PEDOT-C25, third row: C50, fourth row: C75, fifth row: C100). The same measurements were carried out in 0.1 M NaCl, 0.1 M KCl and 0.1 M KPF_6.

10.3. For ~400 nm films

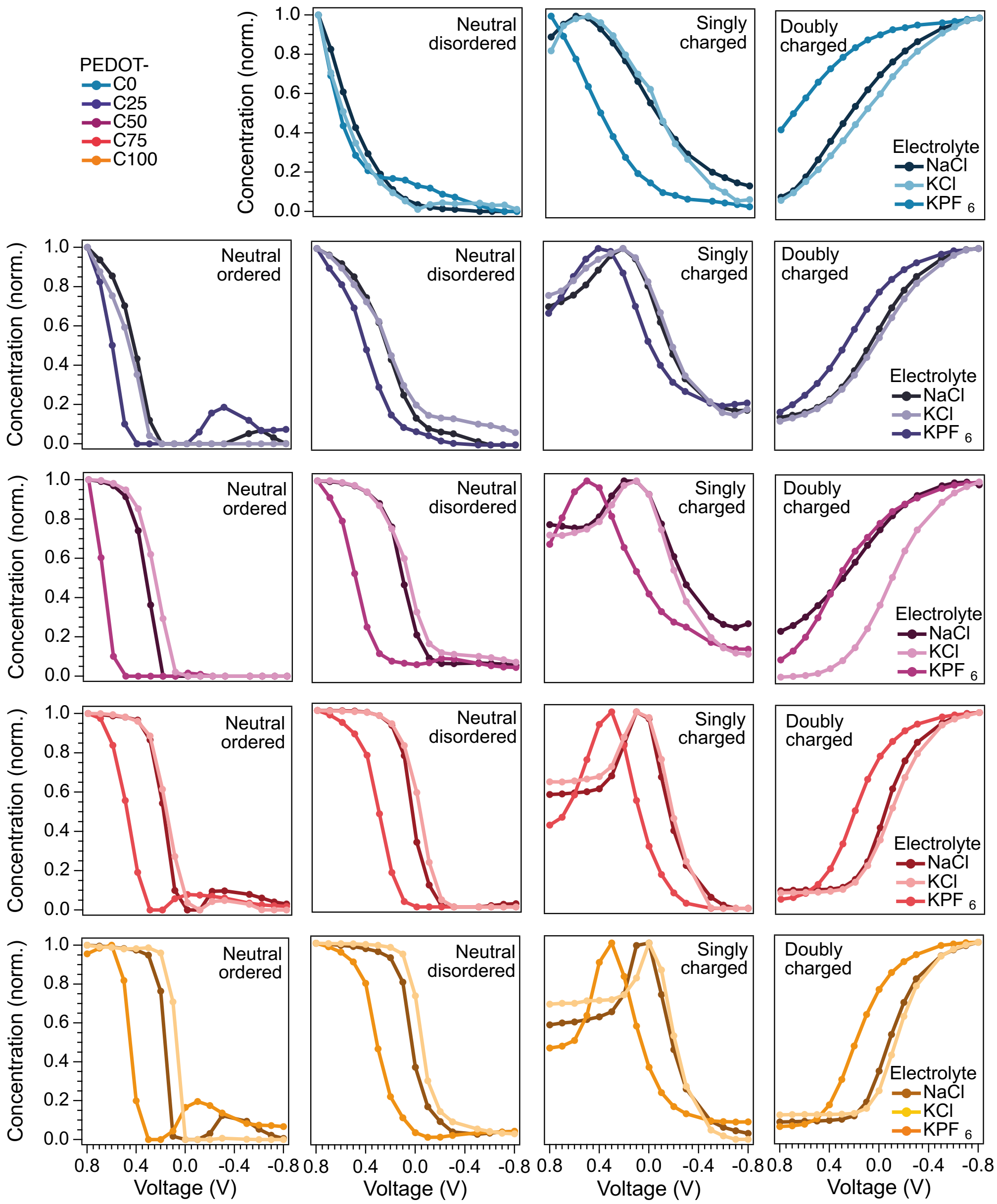

Figure S22. Species concentrations of the ~400 nm films as a function of voltage (first column: neutral ordered, second column: neutral disordered, third column: singly charged species, fourth column: doubly charged species), and the different crown polymers (first row: PEDOT-C0, second row: PEDOT-C25, third row: C50, fourth row: C75, fifth row: C100). The same measurements were carried out in 0.1 M NaCl, 0.1 M KCl and 0.1 M KPF_6.

S.11 Comparable doping level

Due to different redox onsets and redox windows, a comparable doping level was estimated where neutral and singly charged species where in a 1:1 ratio ($Abs_{Neutral}/Abs_{SinglyCharged}$ ~ 1). In the table below, the voltages needed to be applied to reach the same doping level for the different PEDOT-crown derivatives are tabulated.

		~100 nm		~200 nm		~400 nm	
		Ratio N/SC	Voltage (V)	Ratio N/SC	Voltage (V)	Ratio N/SC	Voltage (V)
0.1 M NaCl	PEDOT-C0	1.05	0.4	0.92	0.3	1.06	0.4
	PEDOT-C25	0.90	0.2	1.19	0.2	0.83	0.1
	PEDOT-C50	0.83	0.0	0.82	0.0	0.72	0.0
	PEDOT-C75	0.95	0.0	1.02	0.0	0.85	0.0
	PEDOT-C100	1.02	0.0	1.62	0.0	1.02	0.0
0.1 M KCl	PEDOT-C0	0.99	0.4	1.05	0.4	1.01	0.4
	PEDOT-C25	0.93	0.3	1.12	0.2	0.91	0.1
	PEDOT-C50	0.91	0.0	0.88	0.0	0.92	0.0
	PEDOT-C75	1.09	0.1	1.07	0.0	1.01	0.0
	PEDOT-C100	0.90	-0.1	0.81	-0.1	0.86	-0.1
0.1 M KPF_6	PEDOT-C0	1.00	0.8	0.92	0.8	0.89	0.8
	PEDOT-C25	1.12	0.6	1.11	0.6	0.87	0.3
	PEDOT-C50	1.01	0.4	0.82	0.4	0.86	0.4
	PEDOT-C75	1.19	0.4	1.04	0.4	1.14	0.3
	PEDOT-C100	1.03	0.4	1.33	0.4	1.19	0.3

Table S2. Overview of the comparable voltages where the ratio of neutrals to singly charged species is about 1. The ratios and voltages are listed for the three different used electrolytes (0.1 M NaCl, 0.1 M KCl and 0.1 M KPF_6) and the five different PEDOT-crown polymers.

S12. Doping and dedoping dynamics

The dynamic curves were fitted using a biexponential fit: $f(x) = y_0 + A_1 \exp\left(-\frac{(x-x_0)}{\tau_1}\right) + (y_{max} - y_0 - A_1) \exp\left(-\frac{(x-x_0)}{\tau_2}\right)$, where we can extract the average time constant τ, by scaling τ_1 and τ_2 with their corresponding prefactor A_1 and A_2. Where A_2 is equal to y_{max} – y_0 -A_1. The parameter y_0 is defined by the end concentration, y_{max} by the start concentration and x_0 is the time at y_{max}.

		Initial voltage (V)	Dedoping voltage (V)
0.1 M NaCl	PEDOT-C0	-0.4	0.4
	PEDOT-C25	-0.4	0.2
	PEDOT-C50	-0.4	0.0
	PEDOT-C75	-0.4	0.0
	PEDOT-C100	-0.4	0.0
0.1 M KCl	PEDOT-C0	-0.4	0.4
	PEDOT-C25	-0.4	0.3
	PEDOT-C50	-0.4	0.0
	PEDOT-C75	-0.4	0.1
	PEDOT-C100	-0.4	-0.1
0.1 M KPF_6	PEDOT-C0	-0.4	0.8
	PEDOT-C25	-0.4	0.6
	PEDOT-C50	-0.4	0.4
	PEDOT-C75	-0.4	0.4
	PEDOT-C100	-0.4	0.4

Table S3. Overview of the initial doping voltage and the dedoping voltage, which is the comparable voltages where the ratio of neutrals to singly charged species is about 1. The voltages are listed for the three different used electrolytes (0.1 M NaCl, 0.1 M KCl and 0.1 M KPF_6) and the five different PEDOT-crown polymers.

12.1 Time constants for ~100 nm films

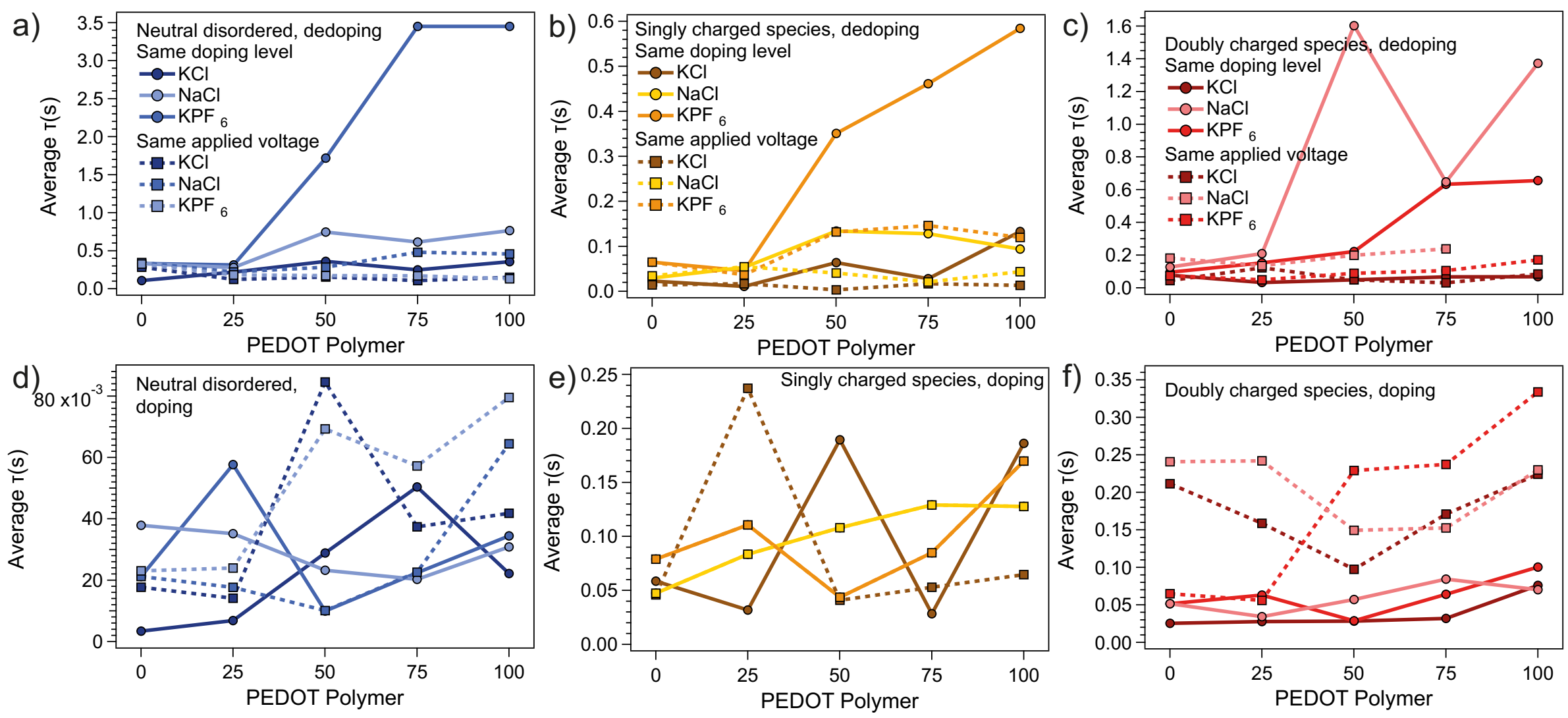

Figure S23. Time constant τ of the dedoping (first row) and doping (second row) process in ~100 nm films extracted from the biexponential fit for the different materials and electrolytes for a, d) disordered neutral, b, e) singly charged and c, f) doubly charged species.

12.2 Time constants for ~200 nm films

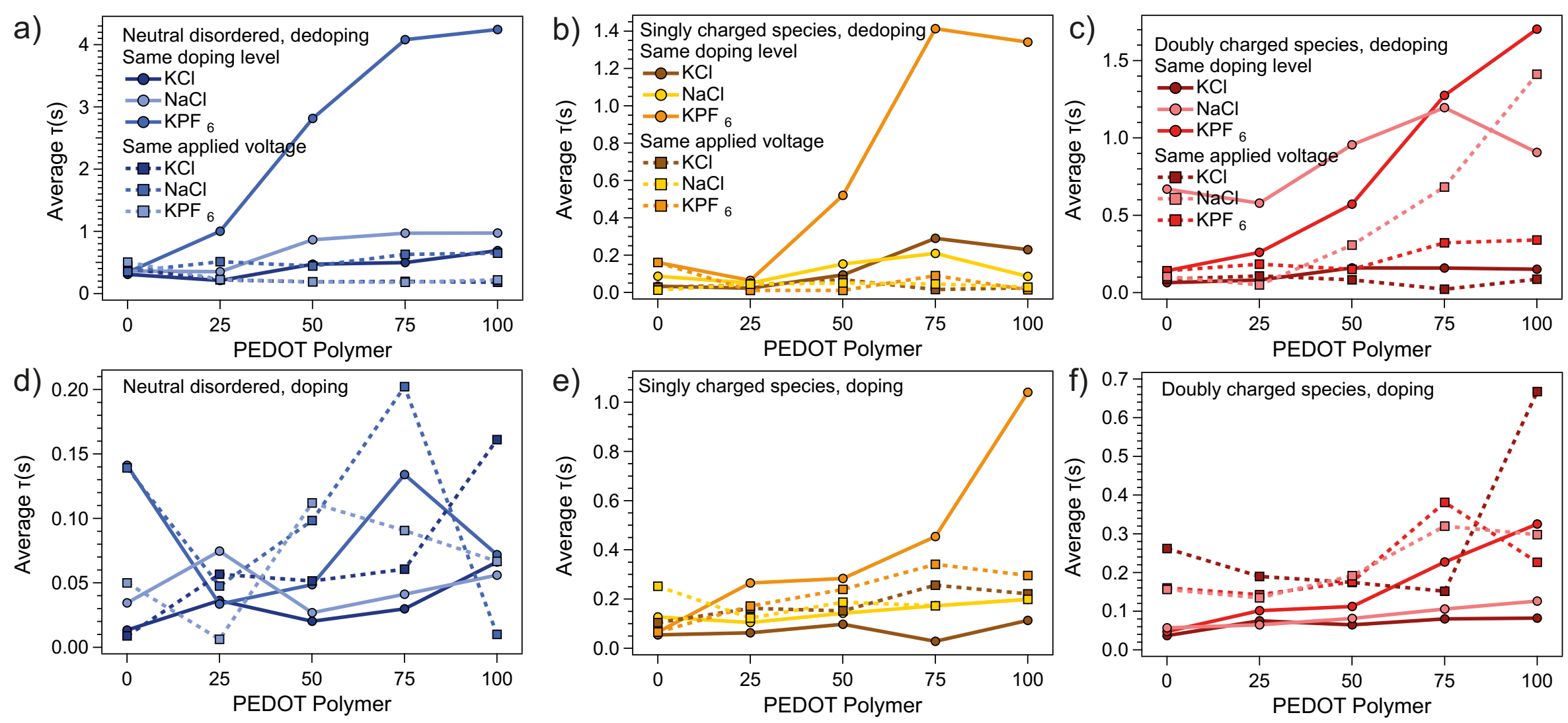

Figure S24. Time constant τ of the dedoping (first row) and doping (second row) process in ~200 nm films extracted from the biexponential fit for the different materials and electrolytes for a, d) disordered neutral, b, e) singly charged and c, f) doubly charged species.

12.3 Time constants for ~400 nm films

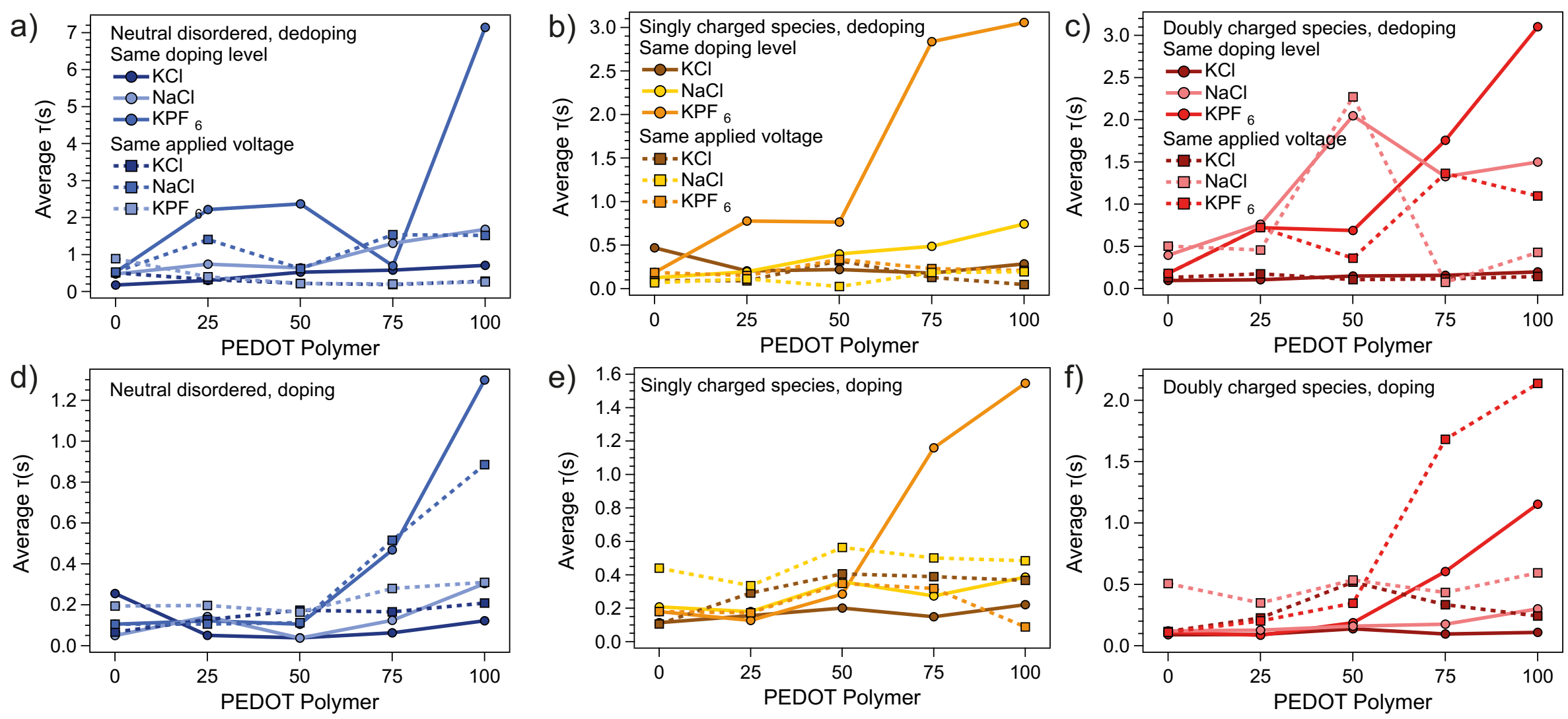

Figure S25. Time constant τ of the dedoping (first row) and doping (second row) process in ~400 nm films extracted from the biexponential fit for the different materials and electrolytes for a, d) disordered neutral, b, e) singly charged and c, f) doubly charged species.

S13. References

(1) Camp, C. H., Jr. pyMCR: A Python Library for MultivariateCurve Resolution Analysis with Alternating Regression (MCR-AR). *J Res Natl Inst Stand Technol* **2019**, *124*, 1-10. DOI: 10.6028/jres.124.018.
